# Label-free peptide profiling of Orbitrap™ full mass spectra

**DOI:** 10.1186/1756-0500-4-21

**Published:** 2011-01-27

**Authors:** Mark K Titulaer, Dominique de Costa, Christoph Stingl, Lennard J Dekker, Peter AE Sillevis Smitt, Theo M Luider

**Affiliations:** 1 Laboratory of Neuro-Oncology and Clinical and Cancer Proteomics, Department of Neurology, Erasmus University Medical Center, Dr. Molewaterplein 50, P.O. Box 2040, 3000 CA Rotterdam, The Netherlands; 2 Academic Medical Center, University of Amsterdam, Meibergdreef 9, P.O. Box 22660, 1100 DD Amsterdam, The Netherlands; 3 Department of Pulmonology, Erasmus University Medical Center, Dr. Molewaterplein 50, P.O. Box 2040, 3000 CA Rotterdam, The Netherlands

## Abstract

**Background:**

We developed a new version of the open source software package Peptrix that can yet compare large numbers of Orbitrap™ LC-MS data. The peptide profiling results for Peptrix on MS1 spectra were compared with those obtained from a small selection of open source and commercial software packages: msInspect, Sieve™ and Progenesis™. The properties compared in these packages were speed, total number of detected masses, redundancy of masses, reproducibility in numbers and CV of intensity, overlap of masses, and differences in peptide peak intensities. Reproducibility measurements were taken for the different MS1 software applications by measuring in triplicate a complex peptide mixture of immunoglobulin on the Orbitrap™ mass spectrometer. Values of peptide masses detected from the high intensity peaks of the MS1 spectra by peptide profiling were verified with values of the MS2 fragmented and sequenced masses that resulted in protein identifications with a significant score.

**Findings:**

Peptrix finds about the same number of peptide features as the other packages, but peptide masses are in some cases approximately 5 to 10 times less redundant present in the peptide profile matrix. The Peptrix profile matrix displays the largest overlap when comparing the number of masses in a pair between two software applications. The overlap of peptide masses between software packages of low intensity peaks in the spectra is remarkably low with about 50% of the detected masses in the individual packages. Peptrix does not differ from the other packages in detecting 96% of the masses that relate to highly abundant sequenced proteins. MS1 peak intensities vary between the applications in a non linear way as they are not processed using the same method.

**Conclusions:**

Peptrix is capable of peptide profiling using Orbitrap™ files and finding differential expressed peptides in body fluid and tissue samples. The number of peptide masses detected in Orbitrap™ files can be increased by using more MS1 peptide profiling applications, including Peptrix, since it appears from the comparison of Peptrix with the other applications that all software packages have likely a high false negative rate of low intensity peptide peaks (missing peptides).

## Background

High throughput Orbitrap™ (Thermo Fischer Scientific, Germany) mass spectrometry (MS) makes it possible to obtain full MS1-spectra and fragmentation-MS2 (MS/MS) spectra of peptides for comparison and identification purposes. The technique can be applied to compare the differences in quantities of proteins in body fluid and tissue samples. The peptides from enzymatic digested proteins are separated on an LC column. During elution, depending on sample complexity 1-100% of separated peptides detected in the spectra of the MS1 scans can be MS2 triggered by the Xcalibur™ instrument software for MS2 fragmentation [1].

The Peptrix application can handle raw Orbitrap™ files as well as MALDI-TOF and MALDI-FT-ICR mass spectra [2-7]. Peptide profiling requires the following basic steps: 1) peak picking from the raw mass spectra; 2) time alignment of the extracted peak masses between different LC runs; 3) aggregation of masses and corresponding intensities of different sample runs on the Orbitrap™ in a peptide profile matrix; and 4) statistical analysis to highlight masses differentially expressed between different groups. A peptide profile matrix, frequently called Peptide Array or PepArray, is created as an output file. Peptide peak intensities are presented in this matrix for all masses detected in every Orbitrap™ measurement. These MS1 masses can eventually be linked to protein identifiers using MS2 sequence information and available protein databases. Table 1 shows a fragment of such a peptide profile matrix. Replicate measurements from a tryptic digested IgG Fab sample are presented as numbers 1, 2 and 3 in the matrix columns, with the retention time and mass of a peptide in the matrix rows, e.g. peptide mass 1239.259 Da eluting at a retention time of 7969.383 s. The three replicate peak intensities measured for the mass 1239.259 Da are given in the matrix cells, e.g. the values 10005, 13333, 19683 in arbitrary units.

**Table 1:** A fragment of a peptide profile matrix or PepArray

MH+	time (s)	Peak intensity in sample 1	Peak intensity in sample 2	Peak intensity in sample 3	Peptide present in sample 1	Peptide present in sample 2	Peptide present in sample 3	Total count of peptides
1238.712	5702.29	30528	25175	23642	1	1	1	3
1238.735	7770.22	12416	9487	7326	1	1	1	3
1238.899	713.267	7848	5629	6229	1	1	1	3
1239.259	7969.383	10005	13333	19683	1	1	1	3
1239.53	4314.73	7110	10243	7283	1	1	1	3
1239.597	8150.09	5207	6428	2798	1	1	1	3
1239.599	4408.91	8264	7158	6992	1	1	1	3
1239.601	7048.683	4542	8373	6982	1	1	1	3
1239.621	1190.17	370540	333496	302810	1	1	1	3
1239.622	6657.29	69391	66874	53379	1	1	1	3
1239.624	5446.54	26198	32726	20632	1	1	1	3
1239.635	4654.07	60855	59416	159055	1	1	1	3
1239.638	6675.558	10973	0	14356	1	0	1	2
1239.64	3143.02	6429	6080	5409	1	1	1	3
1239.642	5808.01	192225	191568	159055	1	1	1	3
1239.692	4271.67	256980	297801	209433	1	1	1	3
1239.734	10051	6161	6481	5449	1	1	1	3
1239.749	7239.35	18034	14470	16265	1	1	1	3
1239.75	6547.471	7043	8459	5901	1	1	1	3
1240.065	5805.98	14427	14851	6499	1	1	1	3
1240.098	5509.82	19378	25322	20168	1	1	1	3
1240.499	2631.25	17863	9718	15101	1	1	1	3
1240.521	4792.05	15576	14008	16506	1	1	1	3

Peptrix is not completely new software, but an extension of already published nameless software. The architecture of Peptrix is described in [7]. The application consists of: 1) a Java™ graphical interface; 2) Mysql database for storage of meta-data; 3) ftp storage of raw data and processed files: and 4) an interface to R for statistical analysis. The software has changed in many aspects with respect to the previously reported version. Firstly, the peptide profile matrix created from LC-MS experiments contains an extra retention time dimension as peptides elute at different time points from the nano-LC column. Peak-picking algorithms over time are implemented combining more Orbitrap™ scans. Time alignment has to be implemented between different LC runs of the sample. Nano-spray ionization from LC-MS also generates multiple charged peptide ions and a different de-isotoping algorithm was implemented than was required for single charged peptides in MALDI-TOF and MALDI-FTICR measurements. Instead of eliminating isotopes from the peak-lists, which is possible in MALDI experiments, mono-isotopes have to be selected from the raw Orbitrap™ spectra by peak-picking algorithms based on expected isotopic intensity distributions.

Other software packages exist for comparing the raw Xcalibur™ MS1 data between samples, possibly converted into mzXML formatted files, e.g. msInspect, MZMine, OpenMS, VIPER, PEPPer, MSight [8-10]. These tools generate peptide profile matrices, in which spectral intensities and retention times of peptide masses from samples belonging to different groups are presented in various ways.

Some of these software packages, such as SuperHirn and SpecArray, did at the time of analysis not run on the Windows Operating System (OS) but only on Linux [9]. Other applications required customized data input formats or connection to pre-filled databases with equipment-dependent retention times for sequenced peptide masses. The Orbitrap™ files contains all the necessary MS1 and MS2 information (for time alignment), and full analysis only requires an internet connection to a protein database interface, e.g. Mascot™, as implemented in Progenesis™. Some applications cannot handle the approximately 1.8 GB big mzXML files, processed by readw.exe version 4.2.1 from the raw files [11]. This can be due to the size of the files causing RAM related issues. Another reason might be that readw.exe generates not entirely correct structurized mzXML files. In some files mzXML closing tags are missing. Readw.exe could not process files larger than 2 GB on our hardware; Intel Xeon W3520 Quad-Core 2.67 GHz processor with 3.5 GB RAM.

**Figure 1:**
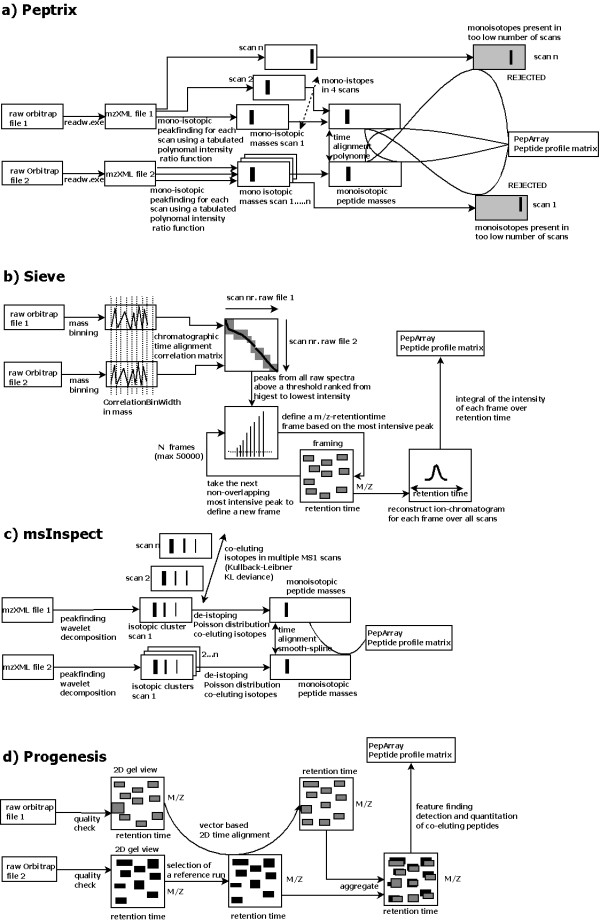
**The (basic) workflow of activities for the compared tools: a) Peptrix, b) Sieve™, c) msInspect, and d) Progenesis™**.

The result of peptide profiling by Peptrix on MS1 spectra were compared with that obtained from a small selection of open source and commercial Windows software packages, i.e. 1) commercial Sieve™ [1]; 2) open source msInspect [12,13]; and 3) commercial Progenesis™ [14]. The aspects compared were: 1) speed; 2) total number of detected masses in the profile matrix; 3) redundancy of masses; 4) reproducibility of number of masses and CV of intensity; 5) overlap of masses between the selected packages; and 6) differences of peptide peak intensities determined by the software packages. The (basic) workflow of activities for the software tools compared - Peptrix, Sieve™, msInspect, and Progenesis™ - is shown in Figure 1.

To compare the four packages, we analyzed three technical replicates of tryptic digested immunoglobulin G (IgG) Fragment antigen binding (Fab) of human serum. We used Peptrix to compare the output of the triplicate measurements from the software packages. Unless important for interpretation of the results, we will not describe how they actually work in terms of algorithms, time alignment, peak selection by isotopic pattern recognition, using peak maxima, features or framing. For these matters, we refer you to the manufacturer's documentation, the comparison study in [9] or the (basic) workflow of activities for the tools compared - Peptrix, Sieve™, msInspect, and Progenesis™ depicted in Figure 1.

As a practical example of Peptide profiling by Peptrix, we present the analysis results of Orbitrap™ measurements of in total 40 micro-dissected tissue samples, 10 spectra of glioma blood vessels, 10 spectra of tissue surrounding the glioma vessels, 10 spectra of normal endothelial vessels, and 10 spectra of endothelial tissue surrounding the normal vessels, previously analyzed by FT-ICR MS [15].

## Methods

The purification of IgG Fab in human serum sample and tryptic digestion of the isolated Fab for MS is described in additional material [Additional Files 1, 2, and 3]. The mass spectrometry measurements are described in Additional File 4.

MS1 Peptide profile matrices of the IgG Fab serum replicate samples were created from raw Orbitrap™ files using Peptrix version 2.4.9, Sieve™ version 1.2, Progenesis™ version 2.0 and msInspect (build 382, 2004-2009) after conversion into mzXML files. The software packages Peptrix and msInspect de-convolute charged masses in isotopic clusters to the mono-isotopic MH^+ ^values. All peptide profile matrices were created with the software package default settings, such as exclusion of single charged masses.

### Mass and retention time window

To prevent software packages recording too much redundant peptide masses and misfits through the use of a retention time window that is too narrow when the peptide profile matrix is being constructed, expected retention time differences of peptides were determined between two consecutive LC runs of replicates.

Based on the maximum expected retention time differences observed in Additional File 5, we used a conservative time window of 5 min [16] (400 frames for msInspect) and mass window of 0.02 Da (10 ppm for Peptrix) for the software packages Peptrix, Sieve™ to produce the four MS1 peptide profile matrices for the three IgG Fab replicates for mainly double and triple charged peptides [Additional Files 6, 7, 8, and 9]. A setting of 50,000 frames was used for Sieve™. Mass and retention time windows could not be set for Progenesis™.

The time window of 5 minutes is used in an additional way in Peptrix. Peptrix has an algorithm that avoids redundancies of peptide masses. When a peptide mass is detected by Peptrix at a specific retention time it is recorded in the Peptide profile matrix. When the same eluting peptide mass is detected again at a later moment within the time window of the previous measurement it is not recorded twice in the Peptide profile matrix. The last measured retention time is used as a new reference point. This process is repeated as long as the mass is measured within the time window of the last measured retention time.

Time alignment in Peptrix was performed using a polynomial fit which follows a theoretical function based on LC-separation theory [17-22] [Additional File 10]. The initial difference in retention time of the bulk of peptide masses is about 1 min but gradually decreases with the retention time of the LC-run to almost 0 after 120 min. In the function, the difference in retention time (dt_R_) decreases with retention time (t_R_) due to a difference in capacity factors (dk_a_) between two runs.

(1) $${\text{dt}}_{\text{R}}={\text{dk}}_{\text{a}}*{\text{t}}_{\text{m}}*\text{exp}\left[-\text{mb}\left({\text{t}}_{\text{R}}-{\text{t}}_{\text{m}}\right)\right]$$

The retention time of the mobile phase (t_m_) was estimated as approximately 2 min, and therefore (t_R _> t_m_). The factor (m) has no dimension. The difference in retention time is also smaller with a larger slope of gradient Acetonitril (ACN) in time (b) with a factor 2 after 120 min [Additional File 5].

### MS2 sequenced and identified masses

Values of peptide masses found in MS1 spectra by peptide profiling were verified using values of MS2 sequenced masses that resulted in protein identifications with a significant score and a Gene Identifier (GI), using the Mascot™ Daemon interface (Matrix Science, UK) [23]. MS2 triggered means that the Xcalibur™ software selects the peak mass in the MS1 spectrum for MS2 fragmentation and sequencing depending on inclusion settings. The MS2 fragmentation does not necessarily result in peptide identifications of proteins with a GI. The quality of the MS2 spectra may be too low to have a significant score from the search engine used. Also sequences of good quality MS2 spectra are sometimes not found in the *in-silico *digested protein databases.

The Thermo Fischer Scientific extract_msn.exe [23] program embedded in Mascot™ Daemon version 2.2.2 [23] interface extracts Mascot™ generic files (MGF) files. The resulting MGF files contain the precursor masses (m/z), their charge states (z), scan identifiers, and peak lists of all MS2 spectra. The MGF files were then sent to the Mascot™ server and the following settings were used for the NCBI human database: tryptic digestion considering 1 possible missed cleavage, variable modification oxidation of Methionine (M) (mass + 15.9994 Da), 10 ppm precursor and 0.6 Da fragment tolerance.

### Comparison matrix

We used Peptrix to compare the matrices from the 4 software packages investigated together with the list of MS2 spectra triggered and MS2 sequenced masses where applicable. It is possible in Peptrix to create a profile matrix of mass-intensity peak-lists from MS experiments [6]. A total of 4 peak-lists containing masses and intensities (1 for each matrix), together with one list of MS2 triggered masses and one list of the MS2 fragmented masses where sequencing succeeded were extracted from the 4 software package peptide profiles. An artificial reference list or grid of 20,275 masses, approximately equal to the number of features in the MS1 profile matrices, was constructed in a mass range between 1,600 and 2,400 Da with fixed distances of 20 ppm between the grid masses. A somewhat greater tolerance than the maximum expected mass inaccuracy of 10 ppm was used to reduce the possibility of slightly different masses being measured in the profile matrices of two different software applications for the same peptide end in two bins.

Peak masses from the generated 6 peak lists: 4 extracted from the matrices produced by the four software packages; the list of MS2 triggered masses; and the list of MS2 triggered masses where fragmentation and sequencing succeeded were matched with the artificial reference list using Peptrix. A mass window setting of plus or minus 10 ppm was used. This forces all the masses between 1,600 and 2,400 Da from the peptide profile matrices to match with at least one of the 20,275 grid points of the reference list. The numbers of overlapping and non-overlapping masses from the software packages were calculated using this constructed comparison matrix (Table 2 and Additional File 11).

**Table 2:** A fragment of the comparison matrix

Mass MH^+^	ms2 triggered	ms2 sequenced	msInspect	Peptrix	Progenesis™	Sieve™
	(for fragmentation)	(proteins found)	Peak intensity (× 10^3^)	Sum peak intensities (× 10^3^)	Peak intensities(× 10^3^)	Peak intensities (× 10^3^)
1741.6671	0	0	0	0	0	0

1741.7007	0	0	473	0	0	652985
1741.7447	1	1	30698	73319	133568	73185
1741.7638	1	0	277640	995497	107051	256383
1741.8094	1	0	3816	222852	14080	0
1741.8397	1	0	41071	146578	58422	78170
1741.8707	1	1	26311	92368	74067	2169410
1741.9122	0	0	3738	19527	6889	0
1741.9355	1	0	202031	95472	564808	273129

1741.9807	0	0	0	0	0	0
1742.008	0	0	1547	0	0	0
1742.0503	0	0	0	0	0	0
1742.0852	0	0	0	0	0	0
1742.12	0	0	0	0	0	0

1742.1472	0	0	0	0	0	101289
1742.193	0	0	0	0	0	45510
1742.2281	0	0	0	2691996	0	0
1742.2594	0	0	0	0	0	0
1742.2909	0	0	1407	69951	0	70092
1742.3208	0	0	0	0	0	3561843
1742.3641	0	0	2474	56134	0	106378
1742.3994	0	0	424	0	0	0

1742.4336	0	0	0	0	0	0
1742.4685	0	0	0	0	0	0
1742.5033	0	0	0	0	0	0
1742.5382	0	0	0	0	0	0
1742.573	0	0	0	0	0	0
1742.6079	0	0	0	0	0	0
1742.6389	0	0	0	0	621012	0
1742.6776	0	0	0	0	0	0
1742.7124	0	0	0	0	0	0

1742.7512	0	0	460	17303	0	60179
1742.7796	0	0	242	5676	0	467398
1742.8157	1	0	1002	67850	82288	43121
1742.8449	0	0	15846	10995	5417	56105
1742.8839	1	1	582	102837	26609	27587
1742.9085	0	0	15849	120678	69898	54135
1742.95	0	0	0	25553	8075	263965
1742.9913	0	0	0	0	0	0
1743.0287	0	0	1005	0	0	0
**1743.0563**	0	0	0	**4248221**	0	0
1743.0959	0	0	0	0	0	0
1743.1307	0	0	0	0	0	0

A total of 4 peak-lists containing masses and intensities (1 for each matrix), together with one list of MS2 triggered masses and one list of the MS2 fragmented masses where sequencing succeeded were matched plus or minus 10 ppm with an artificial reference list or grid of masses with fixed distances of 20 ppm. The comparison matrix contains unoccupied space of MH^+ ^mass values, roughly separated by the lines. The triple charged peptide mass 581.69 Da, which when recalculated to the MH^+ ^value of 1743.0515 Da is only detected by Peptrix.

## Results

### Computation time

Table 3 displays the computation time for Peptrix and the 3 compared software packages. Progenesis™ has the lowest computation time of 1 hour (with 24 MB RAM). The software packages msInspect and Sieve™ need somewhat more time with 2 hours, while Peptrix processes the data in a slightly longer period of 3.5 hours. This is due to: 1) storage of the peak list on an FTP server for every MS1 scan; and 2) the extra comparison steps indicated by the grey boxes in Figure 1a when preparing the matrix. Peptide masses found in at least 4 MS1 scans (file size ~1MB) are also compared with mono-isotopic masses present in less than 4 MS1 scans (file size ~9 MB). These necessary extra comparison steps guarantee reproducibility of peak intensities in the three replicate measurements when working with peak lists.

**Table 3:** Analysis times of the software packages investigated

	Peptrix	Sieve™	msInspect	Progenesis™
Processor	Intel Xeon W3520 Quad-Core 2.67 GHz	Intel Xeon X5472 Quad Core 3 GHz	Intel Xeon 5160 Dual Core 3 GHz	Intel Xeon E5430 Quad Core 2.66 GHz
RAM (Giga Byte, GB)	3.5	3	2	24

Analysis Time (hours)	3.5	2	2	1

### Numbers and reproducibility of peptide masses in the peptide profile matrices

**Figure 2:**
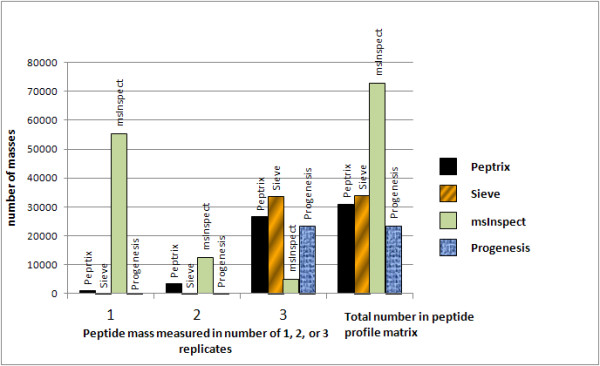
**Histogram representing the number of MS1 peptide masses detected in 1, 2, or 3 technical replicate measurements and the total number of peptide masses in 4 different MS1 software packages**. Ideally all masses should be measured in the 3 replicate measurements of the sample, since the spectra originate from the same sample.

Figure 2 shows a histogram representing the number of peptide masses, recalculated to MH^+ ^values, detected in 1, 2 or 3 technical replicate measurements of the IgG Fab at a specific retention time in MS1 peptide profile matrices produced by the four software packages. Ideally all masses should be measured with the same intensity in the 3 replicate measurements in the sample.

The peptide profile matrices produced by the software packages contain about 20,000 to 70,000 mass-retention time entities (Figure 2). The Peptrix profile matrix contains a total number of 30,986 MH^+ ^masses mainly detected in double or triple charged peak clusters in the spectra [Additional File 6]. 86% of all masses are measured in all three replicates.

Sieve™ displays a larger total number of 33,967 peptide masses in the profile matrix detected in 50,000 frames with charge states > 0, of which about 21,000 masses have charge states 2 or 3 [Additional File 7]. The peptide profile matrix produced by Sieve™ contains peak masses that are nearly present in all 3 replicates for all charge states.

The peptide profile matrix produced by msInspect displays the largest number of masses, i.e. 72,895 masses (Figure 2). The most important reason for the large number of masses and the relative lower overlap in msInspect is that it includes peptide masses that are only present in a few Orbitrap™ MS1 scans. The other software packages use more scans, e.g. Peptrix requires a peptide mass in at least 4 consecutive MS1 scans. In msInspect, most masses occur in one replicate, i.e. 76% of the total number. This is due to the fact that msInspect creates the peptide profile matrix in a sequence-dependent way. It matches a mass in the third replicate if it is already measured in the first and second replicate [Additional File 8]. Therefore, masses that occur only in the second, third or both measurements are not included in the peptide profile matrix.

Progenesis™ measures a low total number of 23,654 masses, of which 19,039 have charge states 2 or 3. Like for Sieve™, nearly all masses measured in all 3 replicates for all charge states (Figure 2). However, this matrix contains redundant peptide masses deviating less than 10 ppm from each other measured at consecutive retention times [Additional File 9].

### Repeated measurement of a single peptide mass in the peptide profile matrices

For practical reasons the retention time was not considered using the comparison matrix in Table 2 for the four MS1 software packages. Undesired misfits would occur when comparing the matrices using time windows. The four software packages show problems with peak tailing of high abundant peaks. Although the intensities in the peak tail are just fractions (≤ 0.1%) of the apex intensities, they still can be detected minutes after the peak, and in extreme cases smear until the end of the run. Such an example is shown for MH^+ ^peptide mass 1502.756993 Da of Ig kappa chain C region (Table 4) with the sequence DSTYSLSSTLTLSK in the supplemental information, which eluted from approximately 85 to 180 minutes. The peptide mass was measured 28 times in the Progenesis™ peptide profile matrix [Additional File 9], about 27 times in the Sieve™ profile matrix [Additional File 7], 8 times in msInspect [Additional File 8], and only 1 time in the Peptrix profile matrix [Additional File 6].

**Table 4:** Peptide masses of in-silico digested Ig kappa chain C region, GI 157838230, either found in the software packages Peptrix, Sieve™, msInspect, Progenesis™ and the Mascot™ Daemon

			Peptrix				Sieve™				msInspect				Progenesis™				Mascot Daemon			
			Peak intensity (× 10^3^)				Peak intensity (× 10^3^)				Peak intensity (× 10^3^)				Peak intensity (× 10^3^)				(GI: gene identifier)			
Mass MH+	#	Sequence	1	2	3	§	1	2	3	§	1	2	3	§	1	2	3	§	1	2	3	§
888.49378	1	EAKVQWK	70	68	60	2	40	37	33	0	17	18	0	4	29	27	26	0	0	0	0	
1502.75844	0	K.DSTYSLSSTLTLSK.A	23172	22659	18733	0	15343	14428	12549	1	10100	9391	8288	3	16869	15721	13663	1	157838230	157838230	157838230	0
1740.87377	0	SGTASVVCLLNNFYPR	55	58	45	1	396	366	320	4	19	21	19	3	118	113	83	1	157838230	157838230	157838230	0
1818.90547	0	VYACEVTHQGLSSPVTK	663	588	465	2	177	182	175	0	155	162	142	0	239	218	193	2	157838230	157838230	157838230	0
1946.02696	0	TVAAPSVFIFPPSDEQLK	29952	10994	24525	2	25191	23771	21825	1	3585	3517	5738	2	83933	78466	73157	2	157838230	157838230	157838230	0
2069.04844	1	SGTASVVCLLNNFYPREAK	17	17	13	0	43	36	28	3	30	0	0	3	6	6	4	1	0	0	0	
2084.05934	1	HKVYACEVTHQGLSSPVTK	2	3	4	0	0	0	0		5	0	0	1	(5	4	4) &	0	157838230	1*	157838230	0
2109.02339	1	DSTYSLSSTLTLSKADYEK	593	576	499	1	201	191	163	0	250	247	221	5	280	265	238	0	157838230	157838230	157838230	0
2135.96873	0	VDNALQSGNSQESVTEQDSK	65032	65514	62998	1	20162	19137	18511	1	33000	3100	0	3	43683	35245	41405	1	157838230	157838230	157838230	0
2323.14995	1	VYACEVTHQGLSSPVTKSFNR	3	7	6	3	13	12	13	4	50	52	36	7	10	11	11	8	0	1*	1*	7
2553.22833	1	DIEMTQSPSSLSASVGDRVTITCR	0	0	0		63	60	52	2	0	0	0		0	0	0		0	0	0	
2677.27	1	VQWKVDNALQSGNSQESVTEQDSK	422	381	329	0	142	134	115	2	279	259	6	7	369	341	304	2	157838230	157838230	157838230	3
3619.70933	1	VDNALQSGNSQESVTEQDSKDSTYSLSSTLTLSK	17	15	13	5	0	0	0		31	34	25	4	68	60	50	1	0	0	0	

# missed cleavage, § mass accuracy in ppm, * MS triggered but no protein matches, & charge 4+ (charge 4+ not included in Peptrix). The spectral intensities of the triplicate measurements of each peptide mass are presented in the columns, except for Mascot™ Daemon. The corresponding GI numbers of the identified proteins by Mascot™ Daemon are presented in the Mascot™ Daemon columns.

Another extreme example is the peptide VYACEVTHQGLSSPVTK with mass MH^+ ^1818.9042 Da of Ig kappa chain C region (Table 4). This peptide eluted between approximately 50 and 130 minutes and the mass was measured 19 times in the Progenesis™ peptide profile matrix [Additional File 9], 10 times in the Sieve™ profile matrix [Additional File 7], about 18 times in msInspect [Additional File 8], and 3 times in the Peptrix profile matrix [Additional File 6].

### Overlap of peptide masses between the MS1 peptide profile matrices

A number of 1,578 Peptrix peptide masses between 1,600 and 2,400 Da differ more than 20 ppm from each other [Additional File 6]. This number represents 27% of the grid points in the comparison matrix. In reality, 38% (> 27%) of the total number of grid points match with a Peptrix single peptide [Additional File 11]. This means that some Peptrix masses within 20 ppm split-up and match with two grid-points. Therefore, the non-matching peptide masses measured between the packages are really significant for 100 * 27/38 ≈ 70%. The other grid points match with more than one mass in the Peptrix peptide profile matrix. Percentages: 36%, 16%, 7%, 2% and 1% of the 5,777 grid points are measured for combinations with 2, 3, 4, 5 and 6 masses of the Peptrix peptide profile matrix respectively. This means that overlap between packages is likely to be overestimated using the comparison matrix grid, not taking the retention time into account.

**Figure 3:**
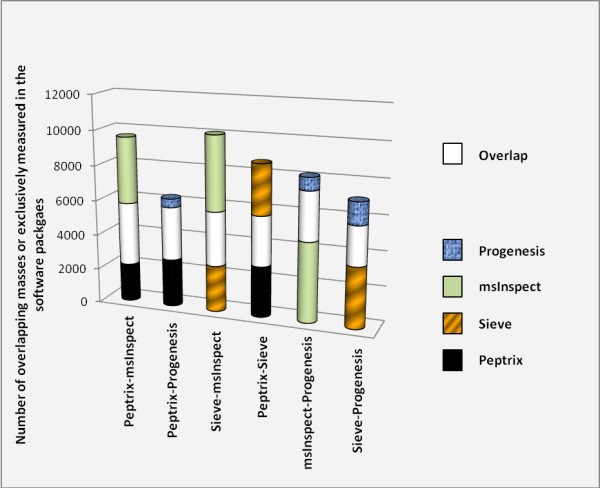
**A pair-wise comparison of overlapping peptide masses, each time between the peptide profile matrices of two MS1 software packages**. A comparison matrix was constructed by Peptrix using an artificial grid of 20275 masses between 1600 and 2400 Da with distances of 20 ppm between the masses. Peak masses from two peptide profile matrices for the software packages Peptrix, Sieve™, Progenesis™ and msInspect were matched with this grid in the comparison matrix, using a mass window with 10 ppm in two directions and a total distance of 20 ppm.

Figure 3 shows the pair-wise overlap between two packages in descending overlap order, using the comparison matrix. The average number of 5,618 masses in the comparison matrix for each package is about four times lower than the 20,275 reference points between 1,600 and 2,400 Da, since the grid in the comparison matrix contains unoccupied space of MH^+ ^mass values (see Table 2 and Additional File 11). The overlap between each time two packages is relatively low with 1/3 of the number of matches with the grid of two software packages together. Most overlap was determined between Peptrix and msInspect, and the least overlap was between Sieve™ and Progenesis™.

**Figure 4:**
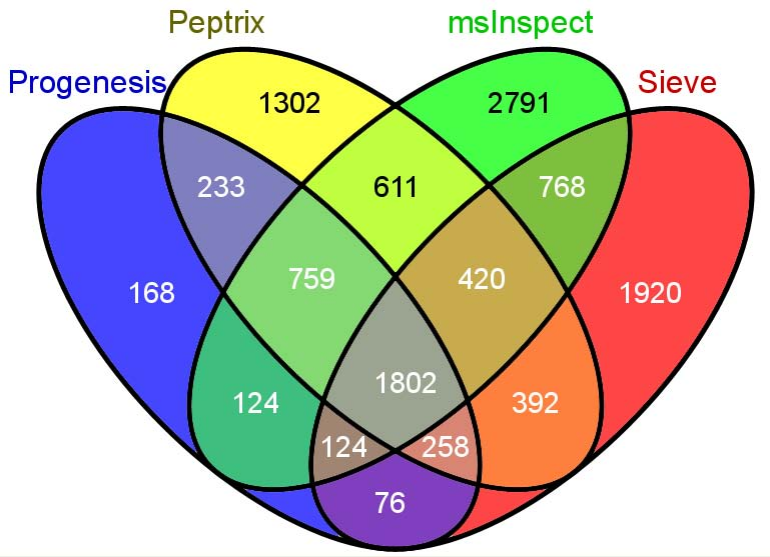
**A 4-way Venn diagram representing the numbers of peptide masses in each profile matrix for Peptrix, Sieve™, Progenesis™ and msInspect, which match between 1, 2, 3 or 4 software packages**. A comparison matrix was constructed by Peptrix using an artificial grid of 20275 masses between 1600 and 2400 Da with distances of 20 ppm between the masses. Peak masses from the individual software package peptide profile matrices were matched with the grids for the comparison matrix, using a mass window with 10 ppm in two directions and a total distance of 20 ppm.

The numbers of matched masses between more than 2 software packages are presented in the 4-way Venn diagram in Figure 4. The number of non-overlapping masses from the software packages is relatively large for Peptrix, Sieve™ and msInspect, i.e. 1,302 for Peptrix, 1,920 for Sieve™ and 2,791 for msInspect, while 160 is measured for Progenesis™. The number of non-matching MH^+ ^peptide masses in Figure 4 increases with the size of the peptide profile matrices (Figure 2). Only a small number of masses (1,802) overlap between all software packages. This number represents approximately 32% of the average total number of 5,618 possible matches with the grid for each software package. If the number of masses present in three applications reflects real masses, the same number represents the number of missing masses, since these masses should be detected by the four software packages. In total 1,561 distinct missing masses MH^+ ^are measured between 1,600 and 2,400 Da; 759+124+258 + 420 (Figure 4). The ratio between detected and not-detected MH^+ ^masses for each software application, irrespective of their accuracy, can be estimated at 1,561:1,802 ≈ 1:1.

### Overlap of MS2 triggered and sequenced masses

**Figure 5:**
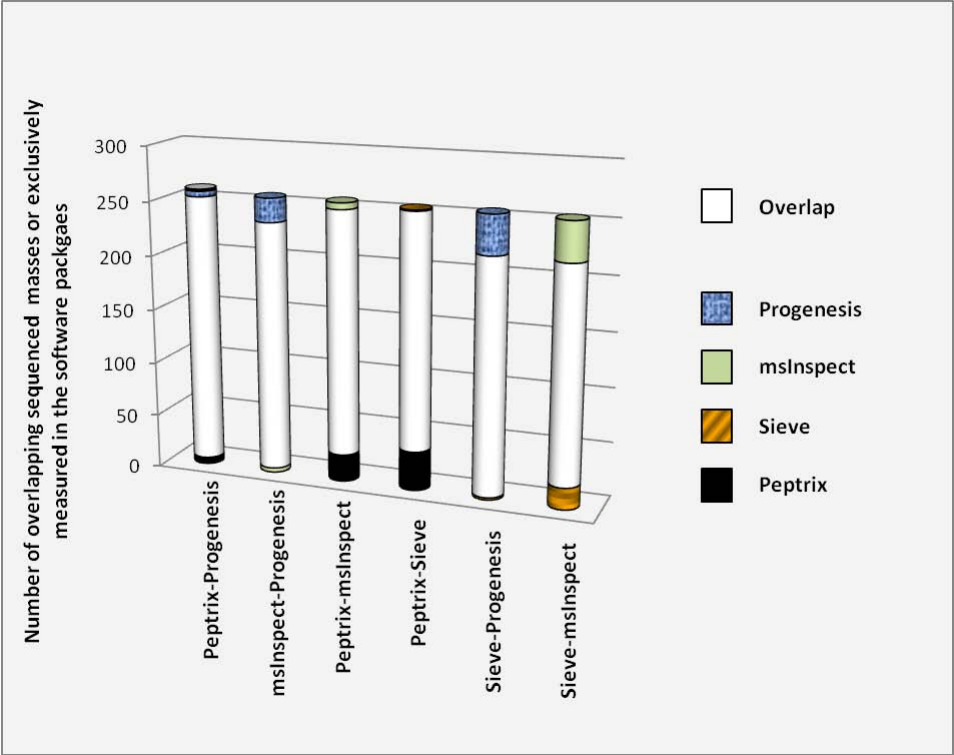
**A pair-wise comparison of peptide masses overlapping with identified protein GI's, each time between the peptide profile matrices from two MS1 software packages**. All masses are sequenced and identified, including the non-overlapping masses in the individual packages. A comparison matrix was constructed by Peptrix using an artificial grid of 20275 masses between 1600 and 2400 Da with distances of 20 ppm between the masses. Peak masses from two peptide profile matrices identified with protein GI's for the software packages Peptrix, Sieve™, Progenesis™ and msInspect were matched with the comparison matrix grid, using a mass window with 10 ppm in two directions and a total distance of 20 ppm.

A sub-selection from the comparison matrix in Table 2 [Additional File 11] was taken for MS2 triggered masses where sequencing succeeded and proteins were identified, using the MGF files from the 3 technical replicates [Additional Files 12, 13, and 14]. Figure 5 shows the pair-wise overlap of MS2 triggered, sequenced, and identified masses between two software packages in descending order of overlap. The most overlap (96%) is measured between Peptrix and Progenesis™, with the least overlap (78%) between Sieve™ and msInspect. We find just 260 MS2 precursors identified in a 3 h gradient between 1,600 and 2,400 Da. One major reason for this relatively low number is that we are working with an IgG Fab fragment sample yielding a lower number of identifications, presumably because quite a proportion of the peptides have unknown sequences not present in the protein database, which means they are MS2 triggered and sequenced, but protein identification succeeded.

**Figure 6:**
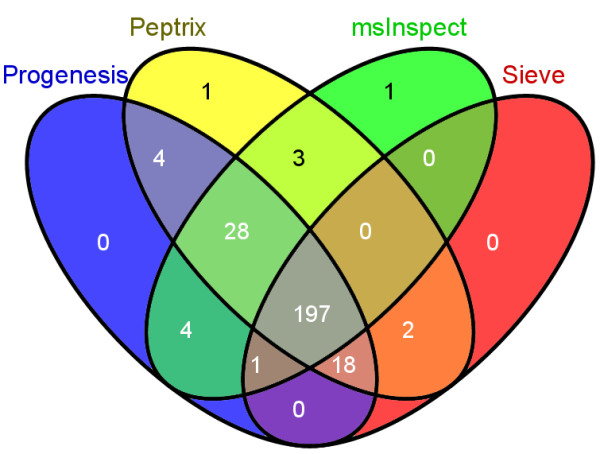
**A 4-way Venn diagram representing the numbers of peptide masses identified with protein GI's in each matrix for Peptrix, Sieve™, Progenesis™ and msInspect, which match between 1, 2, 3, or 4 software packages**. A comparison matrix was constructed by Peptrix using an artificial grid of 20275 masses between 1600 and 2400 Da and distances of 20 ppm between the masses. Peak masses identified with protein GI's from the individual peptide profile matrices for the software packages were matched with the comparison matrix grid, using a mass window with 10 ppm in two directions and a total distance of 20 ppm.

Figure 6 shows the overlap of MS2 triggered and MS2 sequenced and identified peptide masses between all software packages, presented in a 4-way Venn diagram. When comparing identified MS2 precursors from MS2 spectra with a Mascot™ score > 25, the overlap between all software packages is relatively high with approximately 76% (197/260) of the total number of sequenced and identified masses.

### An example of a non-overlapping peptide mass

**Figure 7:**
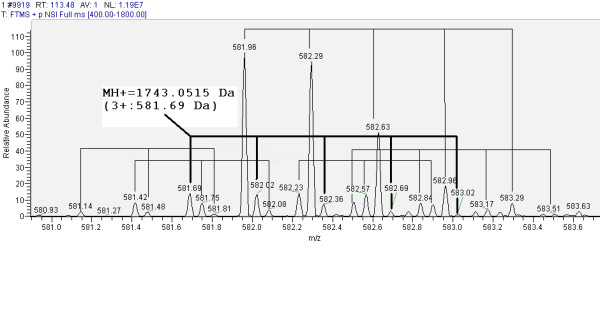
**MS1 spectrum containing a low intensity triple charged peptide peak with mass 581.69 Da, only detected in Peptrix**. The peak at mass 581.69 Da is in an overlapping cluster of triple charged peptide isotopes of approximately 0.33 Da distance. Recalculated to the MH^+ ^value of 1743.0515 Da, the mass is only detected by Peptrix and absent in the peptide profile matrices from the other software packages.

Figure 7 shows an example of an MS1 spectrum for the raw Orbitrap™ file containing a low intensity peak at a triple charged mass 581.69 Da, which when recalculated to the MH^+ ^value of 1743.0515 Da is only detected by Peptrix (Table 2, Additional Files 6 and 11), and absent in the peak lists for the other software packages [Additional Files 7, 8, and 9]. The peak at mass 581.69 Da is in an overlapping cluster of triple charged peptide isotopes. The intensity of the peak is low so the peak is not selected for MS2 sequencing. The isolation width of the mass spectrometer is wide enough to be selected with a more abundant peak close to it, but the intensity of the fragment ions are probably too low to be detected in the resultant MS2 spectrum. The double charged mass for this peptide, 872.03 Da, is present in the spectrum. However, this mass is in a complex overlapping cluster and the charge of this mass cannot be determined. The double charged mass is therefore absent from the peak lists for all packages.

### Differences in peak intensities

Table 4 shows peptide masses MH^+ ^for the *in-silico *digested highly abundant, most frequently sequenced protein Ig kappa chain C region, GI 157838230, found in any of the investigated software packages. The corresponding GI protein number is presented in the Mascot™ Daemon columns of Table 4. It appears that the non-sequenced peptide mass 2553.22833 Da is found exclusively by Sieve™, and is absent from the matrices of the other packages. Conversely, the low intensity MS1 masses 888.49378 and 2069.04844 are found by all software applications in one or more replicates, but are never triggered for MS2.

The peak intensities do not vary much between different replicate measurements in one application, but vary greatly between the investigated applications as show in Table 4. An exception to this is msInspect. Low intensity peaks are not always detected in triplicate by msInspect as was already visible in the histogram in Figure 2, e.g. for mass 2069.04844 Da, which has only one intensity of 30 arbitrary units measured in replicate number 1.

On average, the peak intensity increases in the order: msInspect, Sieve™, Peptrix, Progenesis™. This ranking is not consistent over all peptide masses, however. For example, the high intensity of mass 1502.75844 Da ranks in the order: msInspect, Sieve™, Progenesis™, Peptrix; and the lower intensity of the mass 1740.87377 Da ranks in the order: msInspect, Peptrix, Progenesis™, Sieve™. For the lower peak intensities, with presumably low signal to noise ratios Sieve™ measures a relative high intensity, while for the relative high peak intensities with presumably high signal to noise ratios Progenesis™ and Peptrix measure relative high peak intensities.

**Figure 8:**
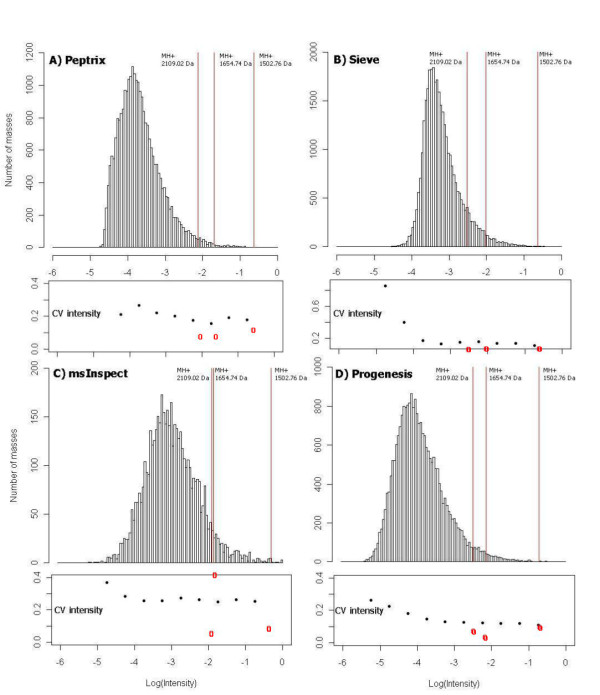
**The distribution of intensity, ^10^log(I/I_max_), and CV's of triplicate measured peak intensities of peptide masses in the peptide profile matrices**. The value ^10^log(I/I_max_) is 0 for the peptide mass with the highest intensity I = I_max _in the peptide profile matrix. The heights of the bars in the histogram represent the number of lower intensity masses with ^10^log(I/I_max_) < 0 of each software package, A) Peptrix, B) Sieve™, C) msInspect, and D) Progenesis™, respectively. The vertical red lines mark the relative intensities, ^10^log(I/I_max_), of 3 peptide masses MH^+^, 2109.02 and 1502.76 Da of Ig kappa chain C region (Table 4), and a mass MH^+ ^1654.74 Da of Ig lambda-1 chain C regions. The intensity distributions of the four software packages align on the logarithm of relative intensities of the 3 peptide masses, approximately at -0.5, -1.9, and -2.2. The maximum in the distribution for each software package represents the number of features just above the signal to noise cut-off. The CV's slightly increase with lower intensity of the peptide peak intensities and more strongly at the signal to noise threshold of peak detection, ^10^log(I/I_max_) < -4.

It is interesting that the relative intensity of features is not preserved amongst the software packages. Figure 8 shows the distribution of intensity, ^10^log(I/I_max_), of triplicate measured peptide masses in the peptide profile matrices of each software package, A) Peptrix, B) Sieve™, C) msInspect, and D) Progenesis™, respectively. The value ^10^log(I/I_max_) is 0 for the peptide mass with the highest intensity I = I_max _in the peptide profile matrix. The heights of the bars in the histogram represent the number of lower intensity masses with ^10^log(I/I_max_) < 0. The vertical red lines mark the relative intensities, ^10^log(I/I_max_), of 3 peptide masses MH^+^, 2109.02 and 1502.76 Da of Ig kappa chain C region (Table 4), and a mass MH^+ ^1654.74 Da of Ig lambda-1 chain C regions. The intensity distributions of the four software packages align on the logarithm of relative intensities of the 3 peptide masses approximately at -0.5, -1.9, and -2.2. The maximum in the distribution for each software package represents the number of features just above the signal to noise cut-off. Sieve™ displays a relative high intensity ^10^log(I/I_max_) of the peak maximum between -3 and -4 in Figure 8A. This agrees with the fact that Sieve™ processes relative high intensity values of low intensity features in Table 4. Peptrix displays the maximum at ^10^log(I/I_max_) just above -4, and Progenesis™ just below -4. The intensity distribution of msInspect displays the maximum at a relative high intensity, due to the relative high intensity of triplicate measured features, 6% of the total measured number of features (Figure 2).

Figure 8 shows the CV's of triplicate measured peak intensities as a function of the relative intensity, ^10^log(I/I_max_), of A) Peptrix, B) Sieve™, C) msInspect, and D) Progenesis™, respectively. The CV's slightly increase with lower intensity of the peptide peak intensities and more strongly at the signal to noise threshold of peak detection; ^10^log(I/I_max_) < -4. The average CV's of triplicate measured peak intensities were: Peptrix 25%; Sieve™ 15%; msInspect 26%; and Progenesis™ 17%.

We calculated the experimental CV of intensity from hand picked triplicate charged mono-isotopic peaks (703.67 Da) and double charged mono-isotopic peaks (1055,01 Da) of mass MH+ 2109,02 in the spectra of 11 scans. The peptide mass MH+ 2109,02 of Ig kappa chain C region, GI 157838230 eluted after 98.4 minutes from the LC column in a time window of 11 scans (1 minute) with ^10^log(I/I_max_) ≈ -2.2. The experimental CV of intensity of the triplicate charged peak was 10% and 14% of the double charged peak with a 4x lower intensity. A CV of 10% was calculated from the intensities of triplicate charged and double charged mono-isotopic peaks together. The CV's were not different calculated from the integral of intensity in time or taking the intensity at the peak maximum in time. The average CV's of triplicate measured peak intensities of mass MH^+ ^2109.02 were; Peptrix 11%; Sieve™ 11%; msInspect 7%; and Progenesis™ 10% (red circles in Figure 8). The CV's agree with the experimental value of 10%.

The CV's of intensity of two other Peptide masses MH+ 1654.74 Da and 1502.76 Da were 9 and 12% for Peptrix, 5 and 10% for Sieve™, 45 and 10% for msInspect, and 5 and 11% for Progenesis™ (red circles in Figure 8). The CV's agree with the experimental value of 10%. The average CV's of the software packages are larger. The larger average CV for Peptrix can be ascribed to matching errors of intensities between the peak lists. An example of such an error is the intensity 10994 in the list of replicate intensities 29952, 10994, and 24525 for mass MH^+ ^1946.02696 in Table 4. The relative high CV of 45% for mass MH^+ ^1654.74 Da for msInspect in Figure 8C can also be ascribed to a wrongly matched intensity.

## Discussion and Conclusions

**Figure 9:**
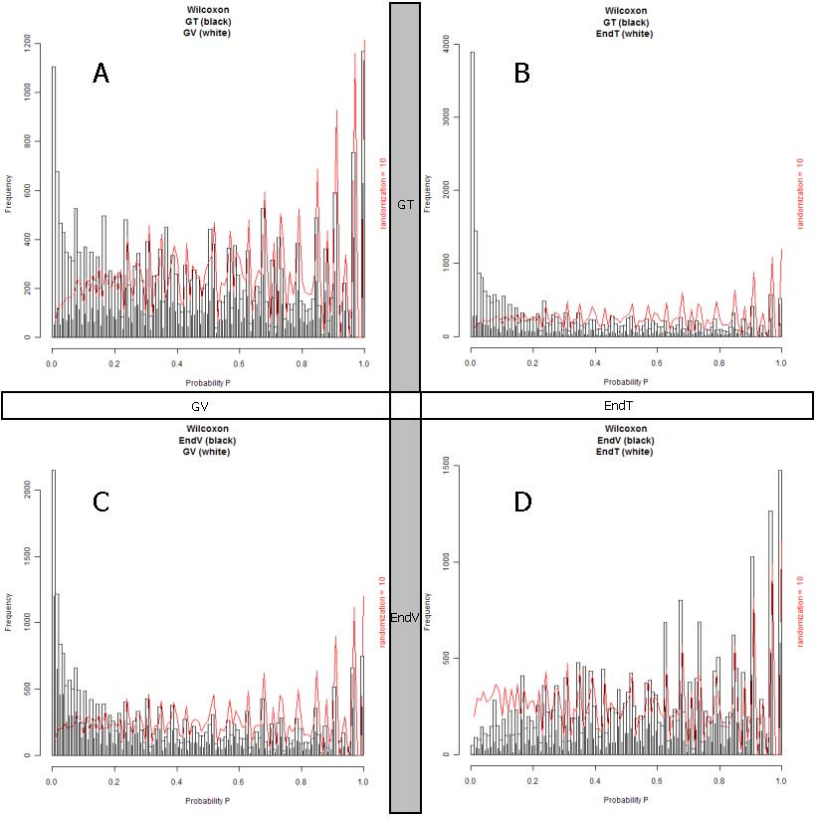
**A practical example of Peptide profiling by Peptrix on a Orbitrap™ data set of in total 40 micro-dissected tissue samples, 10 spectra of glioma blood vessels (GV), 10 spectra of tissue surrounding the glioma vessels (GT), 10 spectra of normal endothelial vessels (EndV), and 10 spectra of endothelial tissue surrounding the normal vessels (EndT)**. A graphical representation of the results of the Wilcoxon-Mann-Whitney tests on peak intensities of masses when comparing the four groups, GV, GT, EndV, and EndT. The peptide profile matrix contains in total 26025 peak masses and 40 samples. The heights of the bars represent the total number of peak masses within a specific p-value interval, whereas the black bar indicates the number of peak masses that were predominantly present in the first mentioned group, and the white bar indicates the number of peaks masses predominantly present in the second mentioned group. The number of peaks masses found for each p-value after 10 times randomization is represented by the red line, which can be viewed as a baseline. Any low p-value bar that exceeds the height of the baseline indicates statistically significant differences in that particular comparison, which means a large number of masses with different peak intensities between both groups. The comparison of GT with GV (A) shows a faint but significant skewing to the left of the p-value range indicates that some peptide masses significant differ in peak intensities between both groups. The same pattern can be seen in (B) but more skewed for the comparison of GT with EndT, which means more significant differences between both groups. Also significant differences can be observed in the comparison of EndV with GV in (C). The comparison of EndV with EndT (D) does not show significant differences.

Peak picking from individual spectra before generation of the profile matrix as implemented in Peptrix has the advantage of parallel processing and scalability to a large number of spectra and distributed computer power. Peptrix, Sieve™, and msInspect run on average computer systems. Comparison of spectral intensities over all samples at once requires a lot of RAM for Progenesis™, which may be a disadvantage with an increasing size of datasets. We determined the reproducibility of the measurements by triplicate LC runs for one sample. We are aware that the number of samples used (3 replicates) does not really reflect an experimental setup for regular proteomics studies, but allows in-depth analysis how these software packages perform technically. The biological replicates used usually far exceed the numbers presented. Therefore we present as a practical example of Peptide profiling by Peptrix, the analysis results of Orbitrap™ measurements of in total 40 micro-dissected tissue samples in Figure 9. Peptrix can analyze the 40 Orbitrap™ raw files of the micro-dissected tissue samples, each of approximately 500 MB in 53 hours, 1 hour and 20 minutes for each file, using a 2.67 GHz Intel Xeon W3520 Quad-Core processor and 3.5 GB of RAM, a relatively low Java memory heap size (XMX) with settings of 1024 Mega Byte (MB).

In particular, the software packages that compare the spectral patterns over all samples directly, such as Progenesis™ and Sieve™, produce very reproducible peak lists with a low CV of intensity as was demonstrated in the histogram of the triplicate measurements for the IgG Fab. A single replicate measurement of a sample in large sample datasets should be sufficient in peptide profiling studies.

When matrices are prepared from peak lists, it is important to also store the masses of rejected peaks into "noise" lists (grey boxes in Figure 1a) to improve reproducibility of the measurements (Figure 2), since low intensity peaks can either just fit or not fit the selection criteria. These additional noise-lists can be used when preparing the peptide profile matrix. First, a peak mass in the list from one sample is matched with the peak mass in the list from another sample. If this mass is not present in the peak list from the other sample, it is searched for in the noise-list from the other sample. An FT-ICR MS example of such an approach was presented in our previous paper [6], and we have extended this approach for LC-MS.

High intensity peptide mass peaks in the MS1 spectra result most frequently in better MS2 fragmentation spectra and lead to more identified proteins after searching for peptide sequences in the protein databases. As expected, the peak picking of the high intensity peaks is more effective since the overlap between the software packages for these sequenced mass lists is higher as been demonstrated in Figures 5 and 6 than for all peptide masses as been demonstrated in Figures 3 and 4. The peptide masses in Figures 3 and 4 include low abundant peptide masses digested from low abundant proteins. It shows that all packages are capable of detecting peptides of high abundant proteins in a reliable way, but that they differ in detection of low concentration peptides. The average overlap for low abundant peptides is 1/2/(1/2+1/2+1/2) ≈ 32% (Figure 3) as approximately 1/2 of the peaks are not found. Peak finding might be especially difficult for low intensity overlapping isotopic clusters in the mass spectra.

MS2 sequencing and protein identification requires accuracy of the mono-isotopic mass. The applications that perform isotopic pattern recognition, such as Peptrix, msInspect, and Progenesis™, show the largest overlap in Figure 5. The software application msInspect has the largest number of non-overlapping masses. In comparing numbers of peptide features detected alone (Figure 2), one could conclude that Sieve™ works the best, followed by Peptrix and Progenesis™. Sieve™ does not perform isotopic pattern recognition. This has the disadvantage that isotopes of a peptide mass can be wrongly assigned as the mono-isotopic mass. This may explain why Sieve™ measures a relative high intensity for the lower peak intensities, with presumably low signal to noise ratios (Table 4). Some features in the Sieve™ profile matrix [Additional File 7] also display non integer values for the charge state, because the real MH^+ ^value is for one reason ore another difficult to calculate (for example overlapping peaks). Sieve™ presumably combines different peptides with different charge states in a single frame (Figure 1b).

False Discovery Rates (FDRs) could be calculated, by comparing the peptide masses in the profile matrices with those from hand-picked peaks in a single MS1 scan, for example scanning 9919 at a retention time of 113.48 minutes. However, in this single scan, hundreds and perhaps even thousands of low intensity peptide features can be detected, making a manual FDR calculation impossible. This indicates that the software packages have likely a high false negative rate (missing peptides).

When comparing the peak lists for *in-silico *digested peptide masses from IgG Fab, it appears that all software packages are capable of extracting almost 100% of the peptide masses, however, with different intensities, and by contrast for msInspect not always in a reproducible way in replicates. Apparently, peak intensities are established by the MS1 software packages investigated in a different way. Peptrix determines the highest intensity of a peptide MH^+ ^mono-isotope mass in a LC elution profile. The intensities of double and triple charged peptides are combined. Sieve™ takes the integral of intensity under the elution curve of a frame (Figure 1b). The software package msInspect determines the highest intensity of an isotopic mass in an isotopic cluster as a function of LC time. This is not necessarily the mono-isotope. Progenesis™ calculates the integral of intensity in two dimensions, in direction of mass and retention time in a 2-D gel view (Figure 1).

## Availability and requirements

The Peptrix java application is freely available and runs on Microsoft Windows 2000 OS or higher. It requires: Readw.exe version 4.2.1, R (R-2.5.1-win32.exe or higher) [24]; Quick 'n Easy FTP Server 3.1 Lite or higher [25]; MySQL 5.0.45 (mysql-5.0.45-win32.zip) or higher database [26]; Java Runtime Environment (JRE) 6 Update 2 or higher (jre-6u2-windows-i586-p.exe) [27]; edtftpj-1.5.5 or higher (edtftpj.jar) [28]; Eclipse IDE for Java Developers - Windows [29]; mysql-connector-java-5.0.7-bin.jar or higher. The source code of Peptrix 2.4.9 is available as a zip file [Additional File 15], as well as the database script [Additional File 16], with detailed installation and running instructions [Additional File 17]. The raw Orbitrap™ files conversion to mzXML formatted files was tested with Readw.exe version 4.2.1 [11] [Additional File 18]. Because the Readw.exe program depends on Windows-only vendor libraries from Thermo, the code for Orbitrap™ data handling will only work under Windows with Thermo Fischer Scientifics' Xcalibur™ software installed. If the Readw.exe program doesn't work properly, download zlib1.dll from Additional File 19. Put zlib1.dll in the c:\windows\system32\directory, and enter "regsvr32 c:\windows\system32\zlib1.dll" in the Windows command prompt (MSDOS box). The latest version 2.5.0 of Peptrix is available as a zip file [Additional File 20].

## List of abbreviations

ACN: Acetonitrile; CSF: Cerebro Spinal Fluid; CV: coefficient of variation; Fab: Fragment antigen binding (of immunoglobulin); FDR: False Discovery Rate; GB: Giga Byte; GI: Gene Identifier; IgG: immunoglobulin G; LC: Liquid Chromatography; MALDI: Matrix Assisted Laser Deionization; MB: Mega Byte; MGF: Mascot™Generic Files; MH^+^: protonated (peptide) mass; MS: Mass Spectrometry; MS1: Full Mass Spectrum; MS2: Fragmentation spectrum (MS/MS) (of a peptide mass); m/z: mass over charge; mzXML: mass over charge eXtensible Markup Language; NCBI: National Center for Biotechnology Information; OS: Operating System; ppm: parts per million (10^-6^); TOF: Time Of Flight; FT-ICR: Fourier Transform Ion Cyclotron Resonance; RAM: Random Access Memory; XMX: maximum memory heap size (of the Java executable); z: charge state

## Competing interests

The authors declare that they have no competing interests.

## Authors' contributions

MKT performed bioinformatics research, designed and programmed Peptrix. DDC performed data analysis. CS and LJD performed the mass spectrometry measurements. All authors read and agreed with the manuscript.

## Supplementary Material

Additional file 1 **Sample preparation**. Click here for file

Additional file 2 **Immobilized Papain instructions**. Click here for file

Additional file 3 **MicroLink™ protein coupling kit instructions**. Click here for file

Additional file 4 **LC separation and Orbitrap™ mass spectrometry measurements**. Click here for file

Additional file 5 **Scatter plot of differences in retention time of potential mass pairs obtained by Peptrix deviating no more than 10 ppm in consecutive LC-runs of replicates of digested IgG Fab**. The difference in retention time is plotted as a function of the retention time in the first run. The initial difference in retention time of the bulk of peptide masses is about 1 min but gradually decreases with the retention time of the LC-run to almost 0 after 120 min, shown by the black polynomial fit. The black points represent retention time differences over two runs of 5 identified peptide masses of the protein Ig kappa chain C region, GI 157838230, i.e. 1502.75844, 1946.02696, 2109.02339, 2135.96873, and 2677.27 Da, varying between 0 and 0.2% of the retention time. Click here for file

Additional file 6 **The Peptrix MS1 peptide profile matrix**. Click here for file

Additional file 7 **The Sieve™ MS1 peptide profile matrix**. Click here for file

Additional file 8 **The msInspect MS1 peptide profile matrix**. Click here for file

Additional file 9 **The Progenesis™ MS1 peptide profile matrix**. Click here for file

Additional file 10 **A theoretical model for retention time differences**. Click here for file

Additional file 11 **A comparison matrix with a grid of 20275 masses between 1600 and 2400 Da with distances of 20 ppm between the masses**. Peak masses from the peptide profile matrices for the software packages Peptrix, Sieve™, msInspect, Progenesis™ and Mascot™ Daemon are matched with the grid, using a mass window with 10 ppm in two directions, and a total distance of 20 ppm. Click here for file

Additional file 12 **The Mascot™ Daemon MS2 export of replicate sample 1**. Click here for file

Additional file 13 **The Mascot™ Daemon MS2 export of replicate sample 2**. Click here for file

Additional file 14 **The Mascot™ Daemon MS2 export of replicate sample 3**. Click here for file

Additional file 15 **Java source code of Peptrix version 2.4.9**. Click here for file

Additional file 16 **Create table script for the MySQL™ database**. Click here for file

Additional file 17 **Installation instructions**. Click here for file

Additional file 18 **The Readw.exe version 4.2.1 (raw to mzXML) converter**. Click here for file

Additional file 19 **The zlib1.dll library**. Click here for file

Additional file 20 **The latest version 2.5.0 of Peptrix**. Click here for file
