# Supplementary material for: Label-free peptide profiling of Orbitrap™ full mass spectra
Source: BMC Res Notes. 2011 Jan 27;4:21. doi: 10.1186/1756-0500-4-21 (PMC3042405; doi:10.1186/1756-0500-4-21)
Supplement: Additional file 1 — Sample preparation. [file 1756-0500-4-21-S1.DOC]

## Sample preparation

*Purification of IgG Fab*

One healthy donor serum sample was obtained from the Sanquin Blood Bank Rotterdam, the Netherlands. This donor (male, age 59 years) did not use any medication. The serum IgG concentration of 9.75 g/l obtained by a turbidity measurement on the Modular P800 equipment (Roche, The Netherlands) was within the normal range (7.0-16.0 g/l).

From this donor, 9 ml venous blood (without additives) was allowed to clot for a period of 1 h at room temperature, centrifuged for 10 min at 2880 g, and stored at 4 °C up to 2 h. Next, the serum of the blood sample was divided in aliquots of 100 μl and stored at a temperature of -80 °C. Of one aliquot, the purified serum IgG fraction was obtained using the Melon Gel IgG purification kit^TM^ (Pierce, USA). The concentration of the purified IgG protein was determined by measuring the extinction coefficient of 1.37 (mg/ml) cm^-1^ at a wavelength of 280 nm in a type ND-1000 spectrophotometer (NanoDrop Technologies, USA).

After the purification step, 400 µl of the purified IgG solution was digested overnight by the enzyme Papain immobilized on Agarose beads according to the manufacturer’s instructions. After digestion, 2700 µl of the Papain digested and purified IgG was concentrated approximately ten times and exchanged in 280 µl 0.1 M sodium phosphate coupling buffer of the MicroLink^TM^ Protein kit (Pierce, USA) using an Amicon Ultra 3K centrifugal filter device (Millipore, The Netherlands).

To separate IgG Fab from crystallizable fragments, Fc, and undigested IgG, protein affinity chromatography was performed using the MicroLink Protein Coupling kit^TM^ (Pierce, USA). In brief, 250 µl Fc fragment specific ImmunoPure anti-human IgG solution was immobilized on MicroLink Protein support of the AminoLink Plus coupling gel spin column at 4 °C overnight with gentle end-over-end mixing. The 200 µl Papain digested purified IgG was loaded onto this column and incubated at a temperature of 4 °C overnight with gentle end-over-end mixing. Finally, the flow-through containing the Fab fragments was collected and analyzed by sodium dodecyl sulfate - poly acryl amide gel electrophoresis, SDS-PAGE.

After separation by SDS-PAGE under reducing conditions, the proteins were fixed for 10 min in 50% methanol, 10% acetic acid, and 40% water (v/v). The protein bands were visualized with the Colloidal Blue staining kit (Invitrogen, The Netherlands) and the surrounding of the gel bands were de-stained for a minimum of 4 hours in de-ionized water.

*Selection of the SDS-PAGE protein band*

The intensity of the protein bands on the stained SDS-PAGE gel was determined by scanning on a Molecular Imager GS-800 Calibrated densitometer with Quantity One^TM^ 1-D version 4.6.5 analysis software (Bio-Rad, The Netherlands). After imaging and analysis of the SDS-PAGE gels, the selected protein bands were manually excised from the gels. The gel plugs were washed with 100 µl ultrapure water, de-stained twice with 200 µl 100 mM NH_4_HCO_3_ in 70% (v/v) acetonitrile (ACN), and 30% water and washed with 200 µl ultrapure water. Liquid was removed and the gel plugs were completely dried for 35 min in a vacuum SPD 1010 Speedvac^TM^ system centrifuge (Thermo Fisher Scientific, USA).

*Tryptic digestion of the isolated Fab for MS*

An aliquot of 20 µl solution containing 0.1 mg/ml trypsin in 3 mM Tris-HCL (pH 8.8) (Promega, The Netherlands) was added to the dried gel plug and the proteins were digested at room temperature overnight. Subsequently, the tryptic peptides were extracted from the gel by adding three times 50 µl of a 0.5% (v/v) formic acid, 30% ACN, and 69.5% water (v/v) solution, mixed using a type 2510 ultrasonic bath (Branson, USA) and the three extraction fluids were pooled. Subsequently, the samples were evaporated for 2 h in a vacuum centrifuge until they were completely dry. For the nano-LC MS/MS analysis, the dried peptides were dissolved in 20 µl of a solution containing 0.1% formic acid, 2% ACN, and 97.9% ultrapure water (v/v), using an ultrasonic bath.
