# Supplementary material for: Label-free peptide profiling of Orbitrap™ full mass spectra
Source: BMC Res Notes. 2011 Jan 27;4:21. doi: 10.1186/1756-0500-4-21 (PMC3042405; doi:10.1186/1756-0500-4-21)
Supplement: Additional file 2 — Immobilized Papain instructions. [file 1756-0500-4-21-S2.PDF]

INSTRUCTIONS

Immobilized Papain

PIERCE
3747 N. Meridian Road
P.O. Box 117
Rockford, IL 61105

20341 0107.1

Number	Description
**20341**	**Immobilized Papain,** 5 ml of settled gel
	Support: Cross-linked 6% beaded agarose
	Supplied: 50% slurry (10 ml total volume) in 50% glycerol, 0.1 M sodium acetate, pH 4.4 containing 0.05% sodium azide
	Activity: 16-40 BAEE units per milligram of papain before immobilization
	Loading: 250 µg papain/ml of settled gel
	**Storage:** Upon receipt store at 4°C. Product is shipped at ambient temperature.

Introduction

Immobilized Papain is convenient for producing Fab and Fc fragments from a variety of IgG species. Papain is a nonspecific, thiol-endopeptidase that has a sulfhydryl group in the active site, which must be in the reduced form for activity. When IgG molecules are incubated with papain in the presence of cysteine, one or more peptide bonds in the hinge region are split, producing three fragments of similar size: two Fab fragments and one Fc fragment. The Fc fragments can be separated from the Fab fragments using immobilized Protein A or by ion exchange chromatography.

The 50,000 dalton Fc fragment is often used for determining specificity of the Fc receptors without interference from antigen binding.[1] Interactions of immunoglobulin molecules with Fc receptors help regulate the physiology of the cell and alteration in Fc receptor function may cause onset or progression of certain diseases. By following the interaction of Fc receptors and immunoglobulins, the phenomenon of cell surface recognition mediation and activation can be studied.[2]

Fab fragments are useful in immunohistochemical studies because the fragments penetrate the tissue better than intact IgG. Also, nonspecific binding from Fc receptors is avoided by using only the Fab portion of the immunoglobulin.

Using Immobilized Papain allows generation of fragments without contamination of the final preparation with enzyme. Immobilization increases enzyme stability against heat denaturation and autolysis and results in longer maintenance of activity. Cleavage can be regulated via digestion time or flow rate through a column. Immobilized Papain is effective with small volumes of dilute proteins and yields reproducible digests.

Procedure Summary

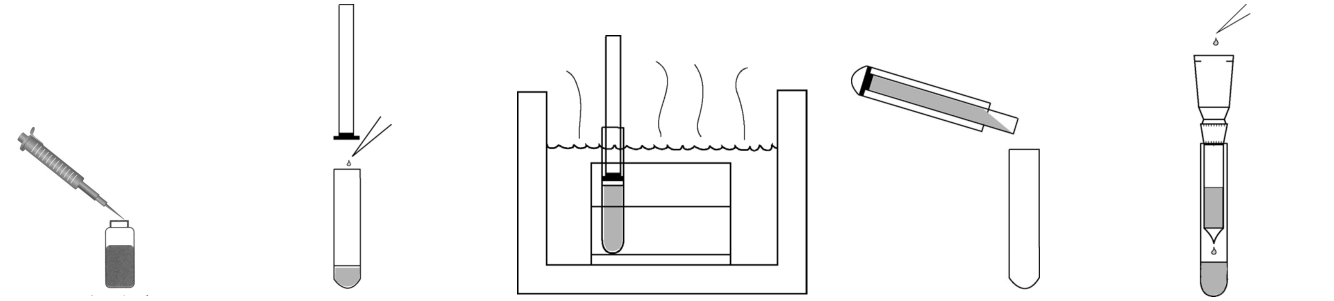

1. Prepare the IgG sample and reagents.

2. Equilibrate the Immobilized Papain.

3. Add the prepared IgG sample and incubate at 37°C.

4. Separate the crude digest from the Immobilized Papain.

5. Separate the Fc fragments from the Fab fragments.

Warranty: Pierce products are warranted to meet stated product specifications and to conform to label descriptions when used and stored properly. Unless otherwise stated, this warranty is limited to one year from date of sale for products used, handled and stored according to Pierce instructions. Pierce's sole liability for the product is limited to replacement of the product or refund of the purchase price. Pierce products are supplied for laboratory or manufacturing applications only. They are not intended for medicinal, diagnostic or therapeutic use. Pierce products may not be resold, modified for resale or used to manufacture commercial products without prior written approval from Pierce Biotechnology. Pierce strives for 100% customer satisfaction. If you are not satisfied with the performance of a Pierce product, please contact Pierce or your local distributor.

Procedure for IgG Digestion to Generate Fab Fragments

This protocol is for digestion of polyclonal mouse and human IgG. Optimization will be required when attempting digestion of IgG from species other than mouse or human. Digestion of various IgG species with non-immobilized and Immobilized Papain is reported in the literature (see Table 1 in the Additional Information Section). Some species of IgG, such as rabbit, are difficult to digest and require a higher ratio of enzyme to substrate (Enzyme: Substrate, 1:10 w/w)[3] than suggested in the following protocol (Enzyme:Substrate, 1:160 w/w), while other species of IgG, such as rat, are easily denatured by this enzyme requiring less enzyme or less time for digestion.

A. Additional Materials Required

- Test tubes or other reaction vessels
- Resin separators (Product No. 69710) or a centrifuge to separate the crude digest from the Immobilized Papain
- Sample Buffer: 20 mM sodium phosphate, 10 mM EDTA; pH 7.0
- Cysteine•HCl (Product No. 44889)
- Shaking water bath capable of maintaining 37°C
- Immobilized Protein A (Product No. 20356) or an ion exchange column to purify Fab or Fc fragments

  Note: Do not use immobilized Protein G because Fabs, as well as Fc fragments, have some affinity for Protein G.
- 10 mM Tris•HCl, pH 7.5

B. Preparation

1. If the IgG is purified and lyophilized, proceed to Step 2. If the IgG is in solution, dialyze against Sample Buffer. Concentrate IgG to approximately 20 mg/ml. Lower concentrations will require a higher enzyme to substrate ratio or a longer digestion time.

   Important: Proper sample preparation is essential for successful fragment generation.
2. Just before use, prepare Digestion Buffer by adding a final concentration of 20 mM cysteine•HCl to the Sample Buffer and adjusting the pH to 7.0.
3. Mix the Immobilized Papain by inversion or gentle shaking to obtain an even suspension. Add 0.5 ml of the 50% Immobilized Papain slurry to a glass test tube or other suitable reaction vessel. To ensure proper gel slurry dispensing, use a wide bore or cut pipette tip.
4. To equilibrate the gel, add 4.0 ml of Digestion Buffer to the gel slurry. Separate the gel from the buffer with a resin separator or by centrifugation. Repeat this wash procedure with another 4.0 ml of buffer. Discard both washes.
5. Resuspend the gel in 0.5 ml of Digestion Buffer.

C. Generation of Fragments

1. Dissolve up to 10 mg of pure lyophilized IgG in 1.0 ml of Digestion Buffer, or add 0.5 ml of the prepared sample to 0.5 ml of Digestion Buffer.
2. Add the 1.0 ml of sample to the tube or vessel containing the Immobilized Papain.
3. Incubate for five hours to overnight in a shaker water bath at 37°C at high speed. Maintain constant mixing of gel during the incubation. For human IgG, incubate for 4 hours using the same conditions.
4. If separating the immobilized enzyme from the digest by centrifugation, add 1.5 ml of 10 mM Tris•HCl, pH 7.5 to the digest before centrifugation, then remove the supernatant, which contains the IgG fragments. If using a resin separator, separate the digest from the immobilized enzyme, then wash the enzyme with the same Tris buffer described above and combine wash with digest.
5. The Fab fragments can be separated from undigested IgG and Fc fragments using an Immobilized Protein A column (Product No. 20356) or ion exchange chromatography.

Additional Information

There are several enzymes that have been used in the fragmentation of immunoglobulins including pepsin, papain, trypsin, elastase, bromelain and ficin. Ficin is used to generate $F(ab')_2$ and Fab fragments from mouse IgG_1, which is typically resistant to other digestion enzymes. Pepsin is most commonly used for the generation of $F(ab')_2$ fragments, and papain, although usually is used to generate Fab fragments, reports indicate that it also can be used to generate $F(ab')_2$ fragments.[4] To prepare $F(ab')_2$ fragments, the papain is first activated with 10 mM cysteine, which is then removed via gel filtration. If no cysteine is present during the papain digestion, $F(ab')_2$ fragments can be generated. These fragments are often inconsistent and reproducibility is a problem. If the cysteine is not completely removed, overdigestion may result.[4]

When using papain to generate Fab fragments, some species of IgG, such as rabbit, are difficult to digest and require a high ratio of enzyme to substrate. Other species of IgG, such as rat, are easily denatured. Digestion of various IgG species with non-immobilized and Immobilized Papain is reported in the literature (Table 1).

Table 1. Digestion of various IgG species with non-immobilized and Immobilized Papain as reported in the literature.

Species IgG	Digestion Method	Purification Method	Reference
**Human**			
Monoclonal IgG_1	Papain	DEAE Cellulose	5
Polyclonal IgG	Crystalline Papain, Papain	DEAE Cellulose, Protein A	6, 7, 8
Anti-*Schistosoma mansoni* soluble egg antigens	Immobilized Papain	Immobilized Antigen	9
Cohn fraction II	Mercuripapain	DEAE	10
**Mouse**			
IgG_{2a} from myeloma	Papain	Protein A	11
Polyclonal IgG	Crystalline Papain, Papain, Mercuripapain	DEAE, Protein A	6, 4
**Rat**			
Monoclonal IgG_1, IgG_{2a}, IgG_{2b}	Mercuripapain	DEAE	2
Polyclonal IgG	Papain	DEAE	6
Polyclonal IgG subclasses	Papain	DEAE Cellulose or Protein A	4
IgG subclasses	Papain or Mercuripapain	Electrophoresis used to monitor digestion	12
**Rabbit**			
Polyclonal IgG	Immobilized Papain, Crystalline Papain, Mercuripapain	Protein A, CM Cellulose	3, 6, 4
**Sheep**			
Antidigoxin	Crystalline Papain	gel filtration, affinity chromatography	13
Polyclonal IgG	Crystalline Papain	DEAE Cellulose	6, 4
**Goat**	Crystalline Papain, Papain, Mercuripapain	DEAE, Protein A, ion exchange	6, 4
**Horse, Cow, Guinea Pig, Chicken**	Crystalline Papain	DEAE	6

Related Pierce Products

44889 **Cysteine•HCl ,** 5 g

69710 **Handee™ Resin Separators,** 25 units

66385 **Slide-A-Lyzer® Dialysis Cassette Kit, 10K MWCO, 0.1-0.5 ml**

66526 **Slide-A-Lyzer® Concentrating Solution,** 10 x 15 ml

20356 **AffinityPak™ Protein A Columns,** 5 x 1 ml settled gel pre-packed columns

44885 **ImmunoPure® Fab Preparation Kit**

44888 **ImmunoPure® F(ab´)2 Preparation Kit**

44880 **ImmunoPure® IgG_1 Fab and $F(ab')_2$ Preparation Kit**

25200-25244 **Precise™ Protein Gels** (see catalog or web site for a complete listing)

References

1. Goding, J.W. (1978). Use of staphylococcal protein A as an immunological reagent. *J. Immunol. Meth.* **20:**241-53.
2. Rousseaux, J., *et al.* (1983). Optimal conditions for the preparation of Fab and F(ab') fragments from monoclonal IgG of different rat IgG subclasses*. J. Immunol. Meth.* **64:**141-6.
3. Coulter, A. and Harris, R. (1983). Simplified preparation of rabbit Fab fragments*. J. Immunol. Meth.* **59:**199-203.
4. Goding, J. (1983). Monoclonal Antibodies: Principles and practice. *Academic Press Inc. London,* 118-122.
5. Raychaudhuri, G., *et al.* (1985). Human IgG1 and its Fc fragment bind with different affinities to the Fc receptors on the human U937, HL-60 and ML-1 cell lines. *Mol. Immunol.* **22(99):**1009-19.
6. Mage, M.G. (1980). Preparation of Fab fragments from IgGs of different animal species. *Meth. Enzymol.* **70:**142-50.
7. Murayama, T., *et al.* (1986). Fc Receptor(s) induced by human cytometalovirus bind differentially with human immunoglobulin G subclasses. *J. Gen. Virol.* **67:**1475-9.
8. Motta-Hennessy, C., *et al.* (1985). Preparation of ^{67}Ga-labelled human IgG and its Fab fragments using desferoxamine as chelating agent*. Eur. J. Nucl. Med.* **11:**240-5.
9. Parra, J.C., *et al.* (1988). Immune responses during human Schistosomiasis mansoni. *J. Immunol.* **140:**2401-5.
10. Nardella, F.A. and Teller, D.C. (1985). Fc intermediate (Fc1), a papain-generated fragment of human IgG, intermediate in charge, molecular weight and cleavage between the Fc and Fc' fragments of IgG. *Molec. Immunol*. **22:**705-13.
11. Goding, J.W. (1978). Use of staphylococcal protein A as an immunological reagent. *J. Immunol. Meth.* **20:**241-53.
12. Rousseaux, J., *et al.* (1980). The differential enzyme sensitivity of rat immunoglobulin G subclasses to papain and pepsin. *Molec. Immunol.* **17:**469-82.
13. Nardella, F.A. and Teller, D.C. (1985). Fc intermediate (Fc1), a papain-generated fragment of human IgG, intermediate in charge, molecular weight and cleavage between the Fc and Fc' fragments of IgG. *Molec. Immunol*. **22:**705-13.

The most current versions of all product instructions are available at *www.piercenet.com*. For a faxed copy, contact customer service (in the USA call 800-874-3723) or your local distributor.

©Pierce Biotechnology, Inc., 4/2004. Printed in the USA.
