# Supplementary material for: Label-free peptide profiling of Orbitrap™ full mass spectra
Source: BMC Res Notes. 2011 Jan 27;4:21. doi: 10.1186/1756-0500-4-21 (PMC3042405; doi:10.1186/1756-0500-4-21)
Supplement: Additional file 3 — MicroLink™ protein coupling kit instructions. [file 1756-0500-4-21-S3.PDF]

INSTRUCTIONS

Thermo SCIENTIFIC

MicroLink™ Protein Coupling Kit

20475

1509.1

Number | **Description**

20475 **MicroLink Protein Coupling Kit,** contains sufficient reagents for 10 coupling reactions using 25-100 µg of antibody or antigen and 20 affinity purifications

Kit Contents:

AminoLink® Plus Coupling Resin Spin Columns, 10 each, columns contain 400 µl of 25% slurry

Coupling Buffer: BupH™ Phosphate Buffered Saline Pack, 1 pack, results in 0.1 M sodium phosphate, 0.15 M NaCl; pH 7.2 when reconstituted with 500 ml of ultrapure water

Quenching Buffer, 50 ml, 1 M Tris•HCl, 0.05% NaN_3, pH 7.4

Sodium Cyanoborohydride Solution (5 M), 0.5 ml, dissolved in 0.01 M NaOH

Wash Solution, 25 ml, contains 1 M NaCl, 0.05% NaN_3

Elution Buffer, 50 ml, pH 2.8, contains primary amine

Microcentrifuge Collection Tubes, 200 each

Storage: Upon receipt store at 4°C. Product shipped at ambient temperature.

Table of Contents

Introduction 1
Important Product Information 2
Material Preparation 2
Coupling Procedure 2
  A. Column Preparation and Protein Immobilization 2
  B. Block Remaining Active Binding Sites 3
  C. Wash and Store the Affinity Column....3
General Procedure for Affinity Purification 3
  A. Form the Resin-bound Complex....3
  B. Elution 4
Troubleshooting....4
References 5

Introduction

The Thermo Scientific MicroLink Protein Coupling Kit allows immobilization of small amounts (25-100 µg) of purified antibody and other proteins directly onto beaded agarose resin to create a permanent affinity support. The Thermo Scientific AminoLink Plus Coupling Resin included in this kit contains aldehyde functional groups that react with primary amines present on antibodies and other molecules. Reductive amination of the resulting Schiff bases forms a stable secondary amine linkage with minimal leakage. Crude sample is incubated with the immobilized protein to form an immune complex. The resin-bound complex is then washed to remove irrelevant material. Finally, the immune complex is dissociated. The purified product can be used for applications such as protein assays, ELISAs, binding studies and Western blotting.

The MicroLink Protein Coupling Kit provides advantages compared to using typical affinity purification methods. Antibody immobilization allows faster and easier immunoprecipitations, enables reuse of the antibody, and results in purified antigen free from antibody contamination. This method features coupling of all primary amine-containing molecules and antibody species and subclasses, in contrast to being limited to antibody species that bind strongly to Protein A or Protein G.

Important Product Information

- The sample solution must not contain amines (e.g., Tris or glycine) as they will quench the reaction. Remove amines before coupling by dialysis using a Slide-A-Lyzer® Cassette (e.g., Product No. 66383) or a Slide-A-Lyzer MINI Dialysis Unit (e.g., Product No. 69576).
- Gelatin or other carrier proteins in the sample solution will compete for coupling sites. Remove gelatin and carrier proteins by performing a Protein A or Protein G purification and subsequent dialysis using PBS. Alternatively, inquire with the antibody supplier if a carrier-/gelatin-free antibody is available.

Material Preparation

Coupling Buffer: Dissolve the dry-blend buffer with 500 ml of ultrapure water. For long-term storage of excess buffer, sterile-filter the solution or add sodium azide to a final concentration of 0.02% and store at 4°C.

Protein Sample: Dissolve protein or peptide to be immobilized with 200-300 µl of Coupling Buffer at 0.5-1 mg/ml. If the sample is not soluble in Coupling Buffer, dissolve it in a suitable buffer (see the Troubleshooting Section). For proteins already in solution, make a 1:1 dilution of the sample with Coupling Buffer or dialyze against the Coupling Buffer.

Note: If the sample is in a buffer that contains primary amines (e.g., Tris or glycine), these compounds will quench the coupling reaction and must be thoroughly removed by dialysis or desalting.

Coupling Procedure

A. Column Preparation and Protein Immobilization

- For all steps requiring mixing the coupling resin with buffer or the sample, gently tap column near the pellet several times until it becomes lose and then gently swirl the column or briefly vortex at low speed.
- Ensure that the resin remains wet at all times.
- Unless otherwise indicated, perform centrifugation steps at 1,000 × *g* for 1 minute.

1. Equilibrate the AminoLink Plus Coupling Resin Spin Column and reagents to room temperature.
2. Loosen the column top cap first and then remove the bottom plug to avoid drawing air into the column. Place column in a collection tube and centrifuge at 1,000 × *g* for 2 minutes.
3. Remove the column cap and insert plug. To suspend resin, add 300 µl of Coupling Buffer or other buffer used to dissolve the sample. Remove plug, place column in a collection tube, centrifuge and discard flow-through. Repeat this step two more times.

   Note: Unless otherwise indicated, perform centrifugation steps at 1,000 × *g* for 1 minute.
4. Plug column and add 200-300 µl of sample (0.5-1.5 mg/ml) directly onto the resin. Replace cap and mix. To determine coupling efficiency, reserve a portion of the sample for use as the starting amount.

   Note: For all steps requiring mixing of the resin, swirl the column gently or briefly vortex at low speed.
5. In a fume hood, uncap column and add 2 µl of the Sodium Cyanoborohydride Solution to the reaction slurry. Replace cap and mix.
6. Incubate column at room temperature for 4 hours. Mix reaction every hour. Alternatively, incubate overnight at 4°C with gentle end-over-end mixing

   Note: For overnight incubation, use a microcentrifuge tube mixer for end-over-end mixing. Make sure the resin is adequately mixing. Adding a final concentration of 0.05% Tween®-20 will help the resin to flow freely in the column.
7. Loosen column cap and remove plug. Place column in a collection tube and centrifuge. To determine coupling efficiency, evaluate the flow-through by protein assay and compare to the starting amount.
8. Uncap column and insert plug. Add 300 µl of Coupling Buffer replace cap and mix. Place column in a collection tube and centrifuge. Repeat this step two more times. Reserve flow-through to evaluate coupling efficiency.

   Note: If the reaction was incubated overnight, then gently invert column 10 times to dislodge any resin remaining in the cap. Loosen column cap and remove plug. Place column in a collection tube and centrifuge. Reserve flow-through to evaluate coupling efficiency. Repeat this step two more times.

B. Block Remaining Active Binding Sites

1. Uncap column and insert plug. Add 300 µl of Quenching Buffer onto the resin replace cap and mix.
2. Remove column cap and plug. Place column in a collection tube, centrifuge and discard flow-through.
3. Repeat Steps 1-2.
4. Plug column and add 200 µl of Quenching Buffer directly onto the resin. Replace cap and mix.
5. In a fume hood, uncap column and add 4 µl of the Sodium Cyanoborohydride Solution to the reaction slurry. Cap column and mix. Incubate reaction at room temperature for 30 minutes mixing every 15 minutes.
6. Remove cap and plug. Place column in a collection tube, centrifuge and discard flow-through.

C. Wash and Store the Affinity Column

Note: For long-term storage (i.e., >2 weeks), add sodium azide to the Coupling Buffer at a final concentration of 0.02%.

1. Plug column and add 300 µl of Wash Solution. Replace cap and mix. Remove cap and plug, place column in a collection tube, centrifuge, and discard flow-through. Repeat this step two more times.
2. Plug column and add 300 µl of Coupling Buffer, replace cap and mix. Remove cap and bottom plug, place column in a collection tube. Centrifuge tube and discard flow-through. Repeat this step two more times.
3. Plug column and add 300 µl of Coupling Buffer along the sides of the column to wash down the resin. Replace cap and store at 4°C.

General Procedure for Affinity Purification

Note: Empirically determine the optimal amount of sample needed and the incubation time for the specific antibody-antigen system being used.

A. Form the Resin-bound Complex

1. Equilibrate the resin containing the immobilized antibody to room temperature.
2. Remove the column cap first and then the plug. Place column in a collection tube, centrifuge and discard flow-through.
3. Plug column and add 200-300 µl of the sample directly onto the resin. Replace cap and mix.
4. Incubate reaction with gentle end-over-end mixing or rocking. Typical incubations range from 2 hours at room temperature to overnight at 4°C.
5. Loosen column cap and remove plug. Place column in a collection tube and centrifuge. Discard or save flow-through for analysis.

   Note: Steps 3-5 may be repeated if additional sample is available and the column's capacity has not been exceeded. Column capacity must be determined empirically.
6. To reduce possible nonspecific interactions (optional) prepare 1 ml of 0.5 M NaCl (dilute the Wash Solution 1:1) containing a final concentration of 0.05% Tween-20. Uncap column, insert plug and add 300 µl of the solution. Replace cap and gently invert column 10 times. Loosen cap, remove plug and place column in a collection tube. Centrifuge tube and discard flow-through. Repeat this step two additional times.
7. Uncap column and insert plug. Add 300 µl of Coupling Buffer, replace cap and gently invert tube 10 times. Loosen cap and remove plug. Place column in a collection tube, centrifuge and discard flow-through. Repeat this step once.
8. Uncap column and insert plug. Add 300 µl of Coupling Buffer, in increments of 100 µl, on the inside surface of the column to wash down the resin. <u>Do not mix the resin</u>. Remove plug, place column in a collection tube, centrifuge and discard flow-through.

B. Elution

1. Plug column and add 100 µl of Elution Buffer along the sides of the column onto the resin. Replace cap and mix. Incubate column at room temperature for 10 minutes.

   Note: Effective elution conditions are dependent on the individual antibody-antigen system and may require optimization.

2. Uncap column and insert plug. Place column in a collection tube and centrifuge. Neutralize the low pH of the eluted fraction by adding 5 µl of 1 M Tris, pH 9.0 or immediately dialyze against PBS using a Slide-A-Lyzer MINI Dialysis Unit (e.g., Product No. 69576).
3. Repeat Steps 1 and 2 as needed. See the Additional Information Section for a method to quickly evaluate elution.
4. Regenerate the resin as soon as possible after elution by washing three times with 300 µl of Coupling Buffer containing 0.02% sodium azide. Store column at 4°C.

Troubleshooting

Problem	Possible Cause	Solution
Low coupling efficiency	Primary amines not completely removed from sample before coupling	Completely remove primary amines by extensive dialysis or desalting
	Protein precipitated during coupling	Check the solubility of the protein in coupling buffer for 4 hours
Protein/peptide is not soluble in Coupling Buffer	Molecule is hydrophobic	Dissolve molecule in Coupling Buffer containing up to 4 M Guanidine•HCl or 20% organic solvent such as DMSO, DMF or CH_3CN
Antigen does not immunoprecipitate	Sample does not contain enough antigen to detect	Verify protein expression and/or lysis efficiency by SDS-PAGE or Western blot analysis of the lysate
	Antibody is not coupled to the resin	Verify that the antibody is coupled to the resin by determining the coupling efficiency
	Antibody is sensitive to low pH and has become inactive during elution	Prepare another antibody-coupled column and use a high-salt, neutral pH elution buffer such as Gentle Elution Buffer (Product No. 21027)
	The antibody-antigen interaction does not elute using acidic conditions	Use a high-salt neutral pH elution buffer, guanidine•HCl, urea, lithium bromide, potassium thiocyanate or nonionic detergents to elute antigen<br><br>**Note:** Using denaturants may cause the antibody to lose activity and, therefore, the antibody-coupled resin cannot be reused

Additional Information

Quick Evaluation of Elution using Pierce Reversible Stain for Nitrocellulose Membranes (Product No. 24580)

1. Dot blot 5-10 µl of each elution fraction onto dry nitrocellulose membrane and let the proteins bind for 2-5 minutes.
2. Wash three times with ultrapure water.
3. Add 25 ml Reversible Protein Stain and shake at room temperature for 30 seconds.
4. Rinse three times with ultrapure water.

Related Thermo Scientific Products

**69715**	**Pierce Microcentrifuge Tubes,** 72 each
**28372**	**BupH Phosphate Buffered Saline Pack,** 40 packs
**21027**	**Gentle Ag/Ab Elution Buffer,** 500 ml
**21004**	**IgG Elution Buffer,** 1 L
**24580**	**MemCode™ Reversible Protein Stain Kit for Nitrocellulose Membranes**
**88013**	**Nitrocellulose Membrane, 0.2 µm,** 7.9 × 10.5 cm, 15 sheets/pkg

References

Bennet, R.M., *et al.* (1988). The production and characterization of murine monoclonal antibodies to a DNA receptor on human leukocytes. *J. Immunol.* **140:**2937-42.

Domen, P., *et al.* (1990). Site directed immobilization of proteins. *J. Chromatogr*. **510:**293-302.

Gersten, D.M., *et al.* (1981). Characterization of immunologically significant unique B16 melanoma produced *in vivo* and *in vitro*. *Proc. Natl. Acad. Sci. U.S.A.* **78:**5109-12.

Harlow, E. and Lane, D. (1988). Antibodies: A Laboratory Manual. Cold Spring Harbor Laboratory: Cold Spring Harbor, New York

Hermanson, G.T., *et al.* (1992). Immobilized Affinity Ligand Techniques. Academic Press, Inc.: San Diego, CA.

Hornsey, V.S., *et al.* (1986). Reductive amination for solid-phase coupling of protein. A practical alternative to cyanogen bromide. *J. Immunol. Methods* **93(1):**83-8.

Horsfall, A.C., *et al.* (1987). Purification of human autoantibodies from cross-linked antigen immunosorbants. *J.Immunol. Methods* **104:**43-9.

Lasch, J. and Koelsch, R. (1978). Enzyme leakage and multipoint attachment of agarose-bound enzyme preparations. *Eur. J. Biochem.* **82:**181-6.

Peng, L., *et al.* (1987). Effect of borohydride reduction on antibodies. *Appl Biochem Biotechnol.* **14:**91-9.

Reeves, H.C., *et al.* (1981). Enzyme purification using antibody cross-linked to protein A agarose: application to *Escherichia coli* NADP-isocitrate dehydrogenase. *Anal. Biochem.* **115:**194-6.

Schneider, C., *et al.* (1982). A one-step purification of membrane proteins using a high efficiency immunomatrix. *J. Biol. Chem*. **257:**10766-9.

Sisson, T.H. and Castor, C.W. (1990). An improved method for immobilizing IgG antibodies on protein A-agarose. *J. Immunol. Methods* **127:**215-20.

Taylor-Papadimitriou, J., *et al.* (1987). Epitopes of human interferon-α defined by the reaction of monoclonal antibodies with interferons and interferon analogues. *J. Immunol.* **139:**3375-91.

Slide-A-Lyzer® MINI Dialysis Unit are protected by U.S. Patent #6,039,871

Tween® is a registered trademark of ICI Americas.

This product ("Product") is warranted to operate or perform substantially in conformance with published Product specifications in effect at the time of sale, as set forth in the Product documentation, specifications and/or accompanying package inserts ("Documentation") and to be free from defects in material and workmanship. Unless otherwise expressly authorized in writing, Products are supplied for research use only. No claim of suitability for use in applications regulated by FDA is made. The warranty provided herein is valid only when used by properly trained individuals. Unless otherwise stated in the Documentation, this warranty is limited to one year from date of shipment when the Product is subjected to normal, proper and intended usage. This warranty does not extend to anyone other than the original purchaser of the Product ("Buyer").

No other warranties, express or implied, are granted, including without limitation, implied warranties of merchantability, fitness for any particular purpose, or non infringement. Buyer's exclusive remedy for non-conforming Products during the warranty period is limited to replacement of or refund for the non-conforming Product(s).

There is no obligation to replace Products as the result of (i) accident, disaster or event of force majeure, (ii) misuse, fault or negligence of or by Buyer, (iii) use of the Products in a manner for which they were not designed, or (iv) improper storage and handling of the Products.

Current versions of product instructions are available at www.thermo.com/pierce. For a faxed copy, call 800-874-3723 or contact your local distributor.

© 2008 Thermo Fisher Scientific Inc. All rights reserved. Unless otherwise indicated, all trademarks are property of Thermo Fisher Scientific Inc. and its subsidiaries. Printed in the USA.
