# Supplementary material for: Label-free peptide profiling of Orbitrap™ full mass spectra
Source: BMC Res Notes. 2011 Jan 27;4:21. doi: 10.1186/1756-0500-4-21 (PMC3042405; doi:10.1186/1756-0500-4-21)
Supplement: Additional file 4 — LC separation and Orbitrap™ mass spectrometry measurements. [file 1756-0500-4-21-S4.DOC]

## Nano-LC separation and Orbitrap^TM^ MS1 and MS2 measurements

LC-MS measurements were carried out on an Ultimate 3000 nano-LC system (LC-Packings-A, Dionex, The Netherlands) online coupled to a hybrid linear ion trap/ Orbitrap^TM^ XL mass spectrometer (Thermo Fisher Scientific, Germany).

Two solvent mixtures were used for LC separation: (I) 97.9% HPLC pure water, 2% ACN, and 0.1% formic acid (Biosolve, The Netherlands); and (II) 19.92% HPLC pure water, 80% ACN, and 0.08% formic acid (Biosolve, The Netherlands).

In each of the 3 consecutive runs, an amount of 5 µl of the digested Fab sample was loaded onto C18 PepMap100 trap columns with a length of 5 mm and an internal diameter of 300 µm (Dionex, The Netherlands). First, 0.1% TFA in water (Biosolve, The Netherlands) was used to trap and desalt the sample/trapping column for a period of 10 min, using a 20 µl/min flow rate. Next, the trapping column was switched in line with a C18 PepMap100 analytical column with a length of 15 cm and an internal diameter of 75 µm (Dionex, The Netherlands), using a flow rate of 300 nl/min.

Over a total period of 180 min, two different slopes of concentration ACN in time were used for the elution of peptides. First, the concentration of ACN was increased by changing the percentage of mixture II in the mobile phase from 0% to 25% in 120 min, resulting in a final concentration of 20% ACN. In a second period of 60 min, the volume fraction mobile phase II was increased to 50%, resulting in a final concentration of 40% ACN. Subsequently, the analytic column was washed and equilibrated for the next gradient cycle.

MS2 acquisition was controlled by Xcalibur^TM^ using data dependent MS2 precursor selection of the five most intensive peaks in each MS1 scan. Precursors selected once were excluded for further MS2 fragmentation by dynamic exclusion for 3 minutes. The exclusion margin was set at 5 ppm (parts per million) in mass. MS1 or MS2 spectra were stored in raw file with 1 s interval time.
