# Supplementary figures and images for: Label-free peptide profiling of Orbitrap™ full mass spectra
Source: BMC Res Notes. 2011 Jan 27;4:21. doi: 10.1186/1756-0500-4-21 (PMC3042405; doi:10.1186/1756-0500-4-21)

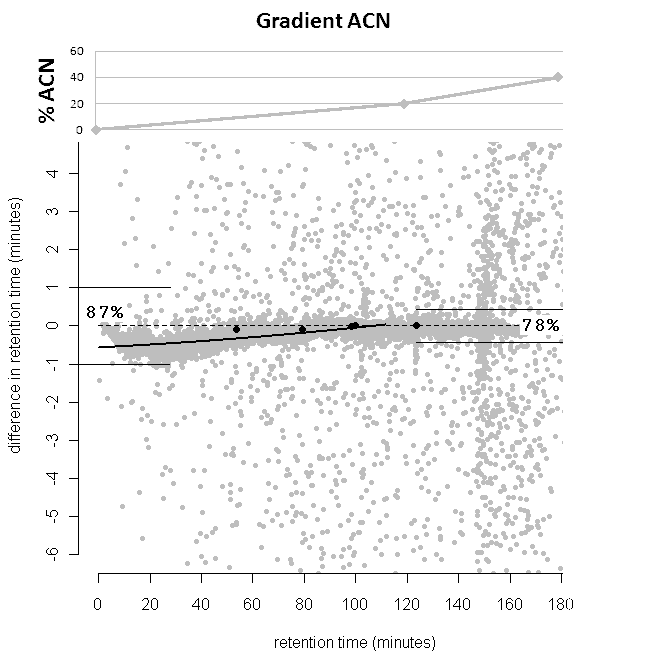

Supplement: Additional file 5 — Scatter plot of differences in retention time of potential mass pairs obtained by Peptrix deviating no more than 10 ppm in consecutive LC-runs of replicates of digested IgG Fab. The difference in retention time is plotted as a function of the retention time in the first run. The initial difference in retention time of the bulk of peptide masses is about 1 min but gradually decreases with the retention time of the LC-run to almost 0 after 120 min, shown by the black polynomial fit. The black points represent retention time differences over two runs of 5 identified peptide masses of the protein Ig kappa chain C region, GI 157838230, i.e. 1502.75844, 1946.02696, 2109.02339, 2135.96873, and 2677.27 Da, varying between 0 and 0.2% of the retention time. [file 1756-0500-4-21-S5.BMP]
