# Supplementary material for: Label-free peptide profiling of Orbitrap™ full mass spectra
Source: BMC Res Notes. 2011 Jan 27;4:21. doi: 10.1186/1756-0500-4-21 (PMC3042405; doi:10.1186/1756-0500-4-21)
Supplement: Additional file 10 — A theoretical model for retention time differences. [file 1756-0500-4-21-S10.PDF]

A theoretical model for retention time differences

Two models have been developed to describe the change in retention time, t_R, of a solute molecule in a mobile phase, A, as function of a gradient in time of volume fraction, Φ_B, of a more- or less polar solvent, B [1]. These are (I) the displacement model, in which solute molecules are displaced at the column surface by the polar solvent molecules, B [1-3]; and (II) the model for the reversed-phase system with a polar mobile phase, A [4, 5]. In the second model, the column capacity factor k (Φ_B) > 0 for a solute molecule as a function of the volume fraction of the organic solvent, B, in the mobile phase is given by

$$k(\varphi_B) = k_a * e^{-m*\varphi_B} \quad (1)$$

where k_a >0 is the capacity factor of the column for the solute molecule in pure water, A, and Φ_B the dimensionless fraction of volume of the less polar solvent ACN, which may vary between 0 and 1, and, m, a dimensionless factor > 0. The retention time of a solute molecule is a function of a time delay after which the solute molecule experiences the gradient of Φ_B due 1) to a dwell time, τ, caused by the dead volume of tubes before the column or start of the experiment; and 2) to the position, z, of the solute molecule in the column, which varies between 0 and the length of the column, L [6]. All formula of this model are well explained [6], except for the complex reciprocal function $d[f^{-1}(\varphi_a(t^*))]\ /\ k(\varphi_a(t^*))$ in the gradient-free period, τ, applying pure water, A, as mobile phase :

$$\frac{d[f^{-1}(\varphi_a(t^*))]}{k(\varphi_a(t^*))} = \text{constant} * t^* \quad (2)$$

where time $t^* = t - (z/u) - \tau$ is the time, t, corrected for the delay or dead time τ and position, z, of the solute molecule in the column, where z varies between 0 and L, u is the speed of the mobile phase, and the quotient $L/u = t_m$ is the retention time of the mobile phase. If no gradient of ACN is applied during the LC separation, $\Phi_B = 0$, the following relation holds [6] :

$$\int_{t=0,z=0}^{t=t_{R,a},z=L} \left\{\frac{d[f^{-1}(\varphi_a(t^*))]}{k(\varphi_a(t^*))}\right\} = \int_{t^*=-\tau}^{t^*=t_{R,a}-t_m-\tau} \left\{\frac{d[f^{-1}(\varphi_a(t^*))]}{k(\varphi_a(t^*))}\right\} =$$

$$\text{constant} * t_{R,a} - \text{constant} * t_m = t_m \quad (3)$$

The retention time, $t_{R,a}$, of a solute molecule in pure solvent, A, is given by [3, 7]:

$$t_{R,a} = (1 + k_a) * t_m \quad (4)$$

Substitution of $t_{R,a}$ in equation (3) by (4) results in

$$\text{constant} = \frac{1}{k_a}, \text{ and consequently } \frac{d[f^{-1}(\varphi_a(t^*))]}{k(\varphi_a(t^*))} = \frac{t^*}{k_a} \quad (5)$$

The integral of the reciprocal function $d[f^{-1}(\boldsymbol{\varphi}_b(t^*))] \ / \ k(\boldsymbol{\varphi}_b(t^*))$ in the gradient in time period, applying a linear increasing volume fraction $\boldsymbol{\varphi}_b$ of ACN with slope b > 0 is:

$$\int_{t^*=0}^{t^*=t_R-t_m-\tau} \left\{\frac{d[f^{-1}(\varphi_b(t^*))]}{k(\varphi_b(t^*))}\right\} = \int_{t^*=0}^{t^*=t_R-t_m-\tau} \left\{\frac{d[\frac{\varphi_b(t^*)}{b}]}{k_a * e^{-m*\varphi_b(t^*)}}\right\} = \int_{t^*=0}^{t^*=t_R-t_m-\tau} \left\{\frac{d[e^{m*b*t^*}]}{m* b*k_a}\right\} \quad (6)$$

The solution of the integrals over gradient free (5) and gradient in time period (6) is [6]

$$t_R = \frac{1}{m*b}\ln\{1 + m * b * [(k_a * t_m) - \boldsymbol{\tau}]\} + t_m + \boldsymbol{\tau} \quad (7)$$

It is not likely that the difference in $\boldsymbol{\tau}$– which, on the sophisticated LC system, is expected to be in seconds – is the cause of the difference in retention time between two experiments. Neither can it explain that we always observe a converging retention time, thus smaller difference in retention time between two experiments as a function of the duration of the LC run. Equation (7) is therefore simplified with $\boldsymbol{\tau} = 0$

$$t_R = \frac{1}{m*b}\ln\{1 + m * b * k_a * t_m\} + t_m \quad (8)$$

The differential of equation (8) is

$$\frac{dt_R}{dk_a} = \frac{t_m}{1+m*b*k_a*t_m} \quad (9)$$

Combination of equation (8) and (9) results in

$$dt_R = dk_a * t_m * e^{-mb(t_R - t_m)} \tag{10}$$

Equation (10) explains that the difference in retention time dt_R drops nearly to zero with an increase of the slope of gradient ACN in time, b. Due to small differences in $\boldsymbol{\tau}$ in s, some difference dt_R from 0 may still remain (equation 7).

References

1. Yoshida T: Prediction of peptide retention times in normal-phase liquid chromatography. Journal of chromatographyA 1998, 811:61-67.
2. Narkiewicz J, Jaroniec M, Borowko M, Patrykiejew A: Dependence of the capacity ratio on the composition of the binary mobile phase in liquid-solid adsorption chromatography Journal of chromatography 1978, 157(1):1-5.
3. Snyder LR: Principles of adsorption chromatography; the separation of nonionic organic compounds. New York: M. Dekker; 1968.
4. Jandera P, Churácek J: Gradient Elution in Liquid Chromatography. Journal of chromatography 1974, 91:207-221.
5. Shinoda K, Tomita M, Ishihama Y: Aligning LC peaks by converting gradient retention times to retention index of peptides in proteomic experiments. Bioinformatics (Oxford, England) 2008, 24(14):1590-1595.
6. Schoenmakers PJ, Billiet HAH, Tijssen R, De Galan L: Gradient selection in reversed- phase liquid chromatography. Journal of chromatography 1978, 149:519-537.
7. Pecsok RL, Shields LD, Cairns T, McWilliam IG: Modern methods of chemical analysis, second edn. New York: Wiley; 1976.
